# Assessment of Food Processing and Pharmaceutical Industrial Wastes as Potential Biosorbents: A Review

**DOI:** 10.1155/2014/146769

**Published:** 2014-07-07

**Authors:** Hanan E. M. El-Sayed, Mayyada M. H. El-Sayed

**Affiliations:** ^1^Mechanical Engineering Department, National Research Centre, Al Bohooth Station, Dokki, Giza 12622, Egypt; ^2^Civil and Environmental Engineering Department, University of Windsor, 401 Sunset Avenue, Windsor, ON, Canada N9B 3P4; ^3^Chemical Engineering Department, National Research Centre, Al Bohooth Station, Dokki, Giza 12622, Egypt; ^4^Chemistry Department, American University in Cairo, AUC Avenue, P.O. Box 74, New Cairo, Cairo, Egypt; ^5^University of Maryland, Baltimore County (UMBC), 252 TRC Building, 5200 Westland Boulevard, Baltimore, MD 21227, USA

## Abstract

There is a growing need for the use of low-cost and ecofriendly adsorbents in water/wastewater treatment applications. Conventional adsorbents as well as biosorbents from different natural and agricultural sources have been extensively studied and reviewed. However, there is a lack of reviews on biosorption utilizing industrial wastes, particularly those of food processing and pharmaceuticals. The current review evaluates the potential of these wastes as biosorbents for the removal of some hazardous contaminants. Sources and applications of these biosorbents are presented, while factors affecting biosorption are discussed. Equilibrium, kinetics, and mechanisms of biosorption are also reviewed. In spite of the wide spread application of these biosorbents in the treatment of heavy metals and dyes, more research is required on other classes of pollutants. In addition, further work should be dedicated to studying scaling up of the process and its economic feasibility. More attention should also be given to enhancing mechanical strength, stability, life time, and reproducibility of the biosorbent. Environmental concerns regarding disposal of consumed biosorbents should be addressed by offering feasible biosorbent regeneration or pollutant immobilization options.

## 1. Introduction

Increased industrial activities resulted in major environmental problems; one of the most challenging is water pollution and the subsequent scarcity in fresh and clean water resources available for current and future generations. Industrial wastewater contains various toxic compounds such as organics, heavy metals, and dyes which could have potential detrimental effect on human beings and aquatic lives. World Health Organization (WHO) recommended the maximum acceptable concentrations for these compounds in water streams. Dyes are one of the most polluted groups as their complex aromatic structure makes them difficult to be biologically degradable [1–3]. They are produced from different industries in large amounts such as textile, paper, leather, food, cosmetics, and pharmaceuticals. It has recently been reported that dye production reached 700,000 tons/year worldwide [4]. Dyes are classified into anionic (direct), cationic (basic), acid and reactive, and nonionic (disperse) dyes [5]. Phenols and phenolic compounds are very toxic and of potential harm to human health. Even at very low concentration (0.005 mg/L), phenols could be of significant odor and taste if present in drinking water [6]. Many industries represent the main sources of phenols such as iron and steel, petroleum, paint, paper and pulp, and pharmaceutics. Nitrophenols and chlorophenols are considered the most hazardous phenolic compounds. Heavy metals are another hazardous group of pollutants. Lead, mercury, cadmium, chromium, copper, and arsenic are examples of the most toxic and carcinogenic elements that can exist in industrial effluents. Metal ions accumulate and their amounts are increased along the food chain due to their nonbiodegradable feature [7, 8]. The main industrial sources of most of these metals are metal finishing and plating, automotive, semiconductor manufacturing, textile, and steel industry.

Removal of such pollutants from different industrial effluents may be achieved* physically, chemically, or biologically*. Physical processes include adsorption, chelation ion exchange, membrane filtration, and coagulation. Chemical methods include oxidation or advanced oxidation and electrochemical treatment, whereas biological methods could be aerobic, anaerobic, or enzymatic [8–10]. These processes known as* conventional* treatment methods have several disadvantages mainly, high energy requirements and capital cost and low efficiency. Conventional treatment methods have been extensively reviewed elsewhere [3, 9, 11–13]. Recently, numerous approaches have been proposed by many researchers for the development of nonconventional and low-cost adsorbents.

Biosorption has become an attractive common technique for many reasons. Being a cost-effective, highly efficient, and easily implemented method made it a successful alternative for the conventional ones [14]. Biosorption as a process may be simply defined as an adsorption on the surface of a compound of a biological origin. Being not limited to only one mechanism and also not restricted to a specific type of pollutant offers a wide variety of applications like pollution control, element recycle, and recovery [10]. A biosorbent may be considered “low-cost” if it satisfied the following conditions: (i) abundance in nature, (ii) requirement for minor or no treatment, and (iii) being a waste material or a by-product from other industries [10]. The challenge here is to choose the appropriate biosorbent that suits the target substance. Selection criteria include, but are not limited to, nature of substance to be adsorbed, mechanism involved in the biosorption process, effectiveness of such biosorbent, cost associated with the whole process, and the possibility of biosorbent regeneration for multiple use cycles. Numerous reviews have been reported on the utilization of agricultural-based, dead or living biomass, and natural adsorbents for the removal of dyes [15–21] and heavy metals [22–28]. Few research studies have focused on the removal of organic pollutants, phenols and phenolic compounds, and pesticides using different biosorbents [29–34].

Disposal of different industrial wastes and by-products is considered a major environmental problem. The cost associated with the waste treatment or disposal, transport, and accumulation may sometimes be the most challenging problem in industry. Such problem increases especially in food industries which produce huge amount of wastes and by-products. Utilizing industrial wastes as low-cost effective biosorbents introduces a bifunctional solution from an environmental point of view. That is to say, treating wastewater effluents with these zero-cost waste materials adds value to these wastes while help solving an important environmental issue.

This paper reviews the state-of-the-art endeavors in utilizing industrial food processing and pharmaceutical wastes as effective low-cost biosorbents for water/wastewater treatment. The aim is to assess the potential of these wastes as biosorbents as well as highlight new options to be further explored and possibilities for improvement. To the best of the authors' knowledge, research dedicated to these particular types of waste has not been reported elsewhere. A comprehensive critical review is presented on (i) the different biosorption techniques and mechanisms, (ii) controlling factors, (iii) equilibrium and kinetics studies, and (iv) recovery and/or pretreatment options. Moreover, concluding remarks will be given at the end along with some suggestions for future work.

## 2. Sources and Applications of Biosorbents

**Figure 1:**
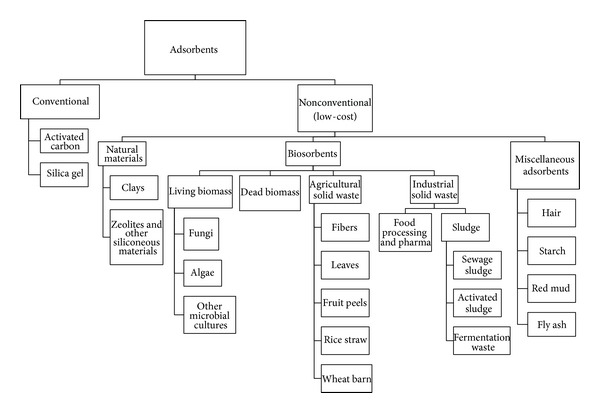
A schematic flow diagram showing the different types of available adsorbents.

Adsorption as a process gained much more attention recently after the use of low-cost adsorbents became so popular especially* biosorbents* [24, 35]. Sources of different types of conventional and nonconventional adsorbents are illustrated in the flow chart in Figure 1.

Biosorbents are a large subclass of low-cost adsorbents that can be subdivided into [36] biomass (dead or living), agricultural wastes, and industrial solid wastes. Dead biomass has been utilized by many researchers as an effective biosorbent for the removal of different pollutants [37, 38]. It has been favored due to its viability to be applied in presence of toxic substances or with shortage of nutrients without this causing appreciable impact on its sorption efficiency. In addition, dead biomass is more readily desorbed than its living counterpart. Living biomasses including fungi [39, 40], algae [41, 42], and other microbial cultures with different strains [43, 44] were also used as low-cost biosorbents. Agricultural-based biosorbents represent a large category of wastes that attracted the attention of many researchers worldwide Utilization of such wastes depended on their local availability. Researchers utilized rice husk and straws [45–48], different nut shells [49–51], fruit and vegetable peels or leaves [52–55], wheat bran [56], chitin and chitosan [57–60], and many other wastes of agricultural origin. Applications of industrial solid wastes in biosorption included the use of sludge whether municipal (sewage) [61] or activated sludge produced from different biological processes [62–66].

Less research has been done on industrial food processing and pharmaceutical wastes despite their huge annual worldwide production. Scarcity of relevant reviews was therefore the main motivation of this current work. Biosorbents from these origins are expected to grow by an annual rate of around 5% in the next few years [67]. For example, food processing waste produced annually in Europe has been reported to be about 2.5 × 10^8^ tons [67]. About 20–60% of the processed fruits and vegetables by volume are generated as waste materials. In the United States, the food manufacturing sector is producing a huge amount of food waste; about 44.3 billion pounds have been reported as per the year 2011 [68].

Utilizing these wastes as biosorbents has been applied in the area of water purification and/or wastewater treatment; previous work in this regard is summarized in Table 1. The table presents type and industrial source of the biosorbent, nature of feed solution, type of targeted sorbate, and operating parameters at which maximum removal was attained. These parameters are pH, temperature, adsorbent dose, and the contact time that was required to reach equilibrium. In addition, mode of operation (batch or column) and its corresponding maximum % removal are included in the table. For column systems, the flow rate (*F*) is also given. Wastes from beverages, tea or coffee, beer or wine, and brewery grains, were used as biosorbents for the removal of many heavy metals and dyes from aqueous solutions [69–83]. Excellent removal efficiencies of up to 99.83% for Cd and 98% for dye sorption onto yeast beer waste [80] and brewery grain wastes [76] were reported. Using beer brewery diatomite waste was recommended for wastewater treatment applications. This is because it released less COD in the industrial wastewater than did the fresh diatomite [81]. Food processing wastes from canning industries were also utilized for the removal of different dyes and heavy metals [84–89]. One particular study was undertaken on real textile wastewater having COD of 426 mg/L, and 97.68% dye removal was achieved when the wastewater sample was spiked with 1 mg/L dye [90]. Studies on the effect of several operating conditions on biosorption performance were also conducted. Results yielded removal efficiencies that reached up to 96.4% for the removal of Cd (II) in case of using okra waste [88] and sugar bagasse waste [89]. Wastes from fruit sources especially orange, mango, and pectin-rich fruits were obtained from juice, jam [91–99], and coconut milk industries [100]. Orange peel [92] and wastes [95] were very effective in removing heavy metals, namely, Pb (II) and Cd (II), with efficiencies of 99.5% and 98%, respectively.

A number of researchers in the Mediterranean countries (Turkey, Spain, Italy, etc.) were interested in olive oil wastes since these countries are among the world's biggest olive producers. All types of wastes from olive oil industry such as pomace, pulp, stones, and milling sludge were used for the sorption of heavy metals and dyes from solutions [101–110]. Particular work [101] reported almost 100% removal efficiency for Cr (VI) from aqueous solutions using olive pomace. Only one study was found to deal with phenol removal [110] using olive oil pomace and removal efficiency was above 90% in both batch and column modes. In another study that investigated the potential of olive oil mill residues as biosorbents for Cu (II), COD release was reduced to 600 mg/L when the biosorbent was washed twice while sorption performance was not affected [104]. Other work utilized different oil industrial wastes such as palm oil waste [111] or sunflower oil waste [112] in the removal of dyes and heavy metals from aqueous solutions.

Commercial activated carbon has been a very common method of adsorption for a long time. Research is now shifted toward using activated carbon derived from various agricultural as well as industrial sources. In the current review, only activated carbons manufactured from food processing wastes are reported. The method of deriving activated carbon (AC) from these wastes will be explained later in the pretreatment section. AC derived from different industrial waste sources such as olive waste cake [113], empty fruit bunch from palm oil mill [114], tea industry [115], and sago waste [116] was successful in biosorption of heavy metals and dyes. In view of the above, it can be inferred that most of the reported studies dealt with the removal of heavy metals and dyes. Clearly, there is lack of work on the removal of other pollutants. One particular research investigated the use of waste cider yeast in removing low concentrations (0.1-0.2 mg/L) of the toxin patulin from apple juice solutions and about 58% removal was achieved [117].

As for pharmaceutical wastes, they are either fungal or bacterial biomass that could be dead or living. Examples of fungal biomass are* Aspergillus niger*,* Pleurotus mutilus*,* Trichoderma reesei*,* Rhizopus arrhizus*,* Rhizopus nigricans,* and* Penicillium chrysogenum*. Bacterial biomass could be produced from antibiotic fermentation such as* Streptomyces* spp. or during production of drugs such as* Streptomyces noursei*,* S. rimosus*, and* S. clavuligerus* or during enzyme manufacture such as the bacillus species of* B. licheniformis* and* S. subtilis*. Plenty of work was done on applications of pharmaceutical wastes as effective biosorbents for contaminant removal from wastewater [118–123]. For heavy metal removal, the waste* Clitopilus scyphoides* (*Pleurotus mutilus*) produced during antibiotic fermentation process was used to remove Cd (II) [120]. A high biosorption capacity of 111 mg/g was obtained within short uptake duration of about 15 min. No pretreatment for the dead biomass was required and the biosorbent was composed predominantly of Ca, Si, and P elements with a total mineral content of 13.5% (w/w). The fungal dead biomass* Aspergillus fumigatus* is also a fermentive waste of antibiotic industry that was utilized in metal biosorption. The sun-dried biomass was pretreated with 5% boiling KOH for 15 min and then thoroughly washed with distilled water till neutral pH was reached. The biosorbent was efficient in removing Cd, Co, Cu, and Ni with the highest efficiency obtained for Cu (72%). More than 90% of metal ions were removed from low-concentration mixtures (0.1 mM). At high concentration of these mixtures, Cu ion was the most competitive among other ions and 70% of which was removed. The fermentation waste mycelium of the fungal biomass,* Aspergillus awamori*, was produced industrially from an enzyme preparation process. It was then utilized for Cr (VI) removal and a maximum removal efficiency of 87% was obtained [124]. The same biomass, after being treated with 0.5 M NaOH, removed a maximum of 35.97 mg/g Cu (II) from aqueous solutions [125].* Tolypocladium* sp. biomass waste was successful in removing Cd, Pb, and Hg [118]. Three different types of fungal biomass from antibiotic industries named Fennel biomass,* Foeniculum vulgare*, which is a medicinal herb, removed 92% of Cd ion at pH 4.3. Maximum biosorption capacities obtained in batch systems were 21, 24, and 30 mg/g at 30, 40, and 50°C, respectively. Biosorption was spontaneous and endothermic. In single-component packed-bed studies, breakthrough and exhaustive capacities were 10 and 40 mg/g, respectively, while 2 and 12 mg/g were the corresponding capacities for multicomponent systems. Capacities dropped to 0.8 and 4 mg/g in multicomponent saline systems. An antibiotic waste composed of a mixture of* Streptomyces fradiae*,* Micromonospora pururea*, and* Nocardia mediterranea* was chemically activated with K_2_CO_3_ to obtain activated carbon that was utilized as a biosorbent for Hg (II) [126].

For the removal of dyes,* Acremonium strictum*,* Acremonium* sp., and* Penicillium* sp. were examined for their potential to decolorize simulated dye baths.* A. strictum* was found to be the most efficient biosorbent with percentage removal of up to 90% in both acidic and neutral conditions. These biomasses were less active compared to* Cunninghamella elegans* which is a biomass known to be an efficient biosorbent that removed 97% of the dye color [121].* Clitopilus scyphoides* (*Pleurotus mutilus*) was also used to remove Basic Blue dye [119] and a biosorption capacity of 200 mg/g was obtained within about 60 min, while Reactive Black 5 (RB5) dye was successfully removed by* Corynebacterium glutamicum* waste produced from lysine fermentation industry [127].

## 3. Operating Factors Influencing Biosorption

Holistically, the behavior and performance of biosorption are affected by the physical and chemical characteristics of each of the biosorbent and sorbate; in addition to the process operating conditions. Biosorbent and sorbate characteristics include composition, structure, type of charged and uncharged functional groups, and particle size. It was also reported that in biomass sorbents, the composition of the cell wall influences both sorption uptake capacity and selectivity [88, 128].

Operating conditions are instrumental biosorption controlling parameters which include pH, temperature, initial sorbate concentration, biosorbent dose, contact time, agitation speed, sorbent particle size, mode of operation, and competition from coions. These operating parameters will be further discussed in more detail.

### 3.1. Solution pH and Ionic Strength

The pH of sorbate solution plays a vital role in the biosorption process since it influences the charge on the biosorbent functional groups and the dissociation of these groups on the active sites. It also affects sorbate solubility and its degree of ionization. The effect of pH on both uptake capacity and percentage removal was investigated by numerous workers. For heavy metal sorption, it was found that the increase in pH increases uptake capacity of heavy metals such as Cd, Pb, Ni, Cu, and Zn in both the acidic and the neutral range (pH 2–7). The rate of increase under highly acidic conditions (pH 2–4) was mostly higher than that observed at milder acidic conditions (pH 4–6) [6, 72, 84, 100–102, 104, 108, 125, 129, 130]. Under basic conditions (pH > 7), heavy metal uptake decreased with pH [100, 101, 108]. Under severe acidic conditions, the very low reported uptake capacities were attributed to the fact that H^+^ ions compete with the metal ions on the active sites [72, 101, 126] which indicates that biosorption is governed by electrostatic interactions under these conditions. Increase in pH also increased the percentage of metal removal under acidic and neutral pHs [97, 105, 106, 126] and decreased the removal under basic conditions [97]. However for the pharmaceutical waste mycelium of the* Aspergillus awamori*, the increase of pH from 2.0 to 4.0 decreased the removal efficiency of Cr (VI) by about 50% [122]. At basic pHs, biosorption uptake decreases owing to metal precipitation which leads on the contrary to increase in metal removal from solution by a possible combined microprecipitation-biosorption mechanism [105–108]. In case of dyes, the behavior of sorption uptake and percentage removal with pH varies according to the type of charge. For cationic dyes such as Methylene Blue (MB) and Basic Blue 41, both removal and uptake are directly proportional to pH [78, 83, 91, 98, 119] and vice versa for anionic dyes such as Acid Green (AG), Acid Red 57, Reactive Red (RR 198), and Victazol Orange 3R dyes [70, 86, 91, 99]. At high pH, the uptake and removal of cationic dyes increase due to attractive forces between the positively charged dye and the negatively charged functional groups on the biosorbent. One study on the removal of phenols by olive pomace showed that removal efficiency is enhanced by increasing pH [110].

Very limited studies were conducted on the effect of ionic strength where the presence of NaCl [105, 123] and perchlorate salts [105] significantly reduced biosorption due to competition between the salt ions and the sorbate ions on the active sites.

### 3.2. Initial Sorbate Concentration

The increase in the initial concentration of the sorbate acts as a driving force to overcome the mass transfer resistance and hence increase the uptake. This behavior was reported for both heavy metals and dyes [83, 98, 110, 125, 126, 130]. In one study dealing with Hg sorption onto desiccated coconut waste, the concentration-uptake correlation was linear [100].

The percentage removal, on the other hand, was found to decrease with increasing in concentration for the heavy metals Cd, Zn, and Ni onto tea, olive cake wastes, and wine processing sludge, respectively [72, 76, 119] as well as for Cr (VI) and Basic Blue 41 dye onto mycelium of* Aspergillus awamori* and antibiotic fungal waste, respectively [108, 124]. The same behavior was encountered by Methylene Blue dye onto both fresh malted sorghum mash waste and mango seed kernel powder [83, 98] and by Pb onto both activated carbons from sago waste and peach/apricot stones. With the latter adsorbent, removal was almost constant at very low concentrations of Pb (5–100 ppm) [97, 116]. With higher initial concentrations, higher equilibrium concentrations in the solution were obtained possibly due to saturation of active sites and this, in turn, decreased the removal efficiency. In addition, the increase in initial concentration decreased sorption rate since it probably reduced diffusion across the boundary layer. However, a different behavior was observed for Acid Green (AG) dye sorbed onto spent brewery grains where the removal initially increased by virtue of the high concentration gradient driving force and then dropped owing to saturation of the sorption active sites [70]. Furthermore, with investigating the sorption of Cd (II), Pb (II), and Cu (II) onto orange peels the dissociation constant for the biosorption interaction decreased exponentially with increase in concentration [101] indicating stronger binding.

### 3.3. Biosorbent Dose

Generally as the biosorbent dose increases, the number of available active sites increases and thus consequently enhances the removal [70, 72, 86, 97, 98, 102, 110, 115, 119, 120, 124, 130]. On the other hand, the uptake capacity decreases probably due to decrease in surface area that might be a result of having some of the sorption sites aggregated and overlapped [100, 102, 108, 129]. In a few cases, the removal reaches a peak value then declines [82, 101, 119] and this could be due to saturation of active sites.

### 3.4. Temperature

The effect of temperature becomes important when dealing with wastewater effluents that are discharged at high temperatures due to processing. For endothermic reactions, biosorption uptake capacity and removal efficiency increased with temperature due to increase in surface activity and hence availability of more active sites [76, 80, 83, 98, 113, 120, 123, 124, 129] and vice versa for exothermic reactions [70, 86, 91, 100]. Sorption rate for endothermic reactions was also enhanced as temperature increased and it followed Arrhenius equation [76, 106].

### 3.5. Particle Size of The Sorbent

In most of the reported studies, the initial rate of sorption was rapid and it decreased gradually till it reached an approximately constant value [70, 101, 106]. This shows that binding mostly occurs on the solid surface and that film and pore ion diffusion are not significant or have very fast rates. In cases where this did not hold true, either film (boundary layer) or pore (intraparticle) diffusion or a combination of both was the rate limiting step [91, 100, 120, 123] When the governing mechanism was the surface reaction, decrease in particle size was shown to improve the uptake [110, 119] due to increase in surface area. In addition, the decrease in particle size enhances diffusion and sorption rates since it reduces intraparticle diffusion [108]. In case of very porous biosorbents like pectin wastes, it was found that particle size had no significant effect on Cr (III) sorption since the external surface area does not contribute much to the total surface area [102].

### 3.6. Agitation Speed

The speed of agitation was found to enhance removal efficiency by reducing mass transfer resistances but only up to an optimal limit above which efficiency drops probably due to biomass fragmentation [82, 103, 119].

### 3.7. Mode of Operation

The operational mode influences uptake and % removal because dynamics of batch systems are different from column dynamics. In most studies, dynamic capacity was lower than its batch counterpart; and the same held true for % removal [100–102, 104, 131]. Column dynamics vary with column dimensions and flow rate. The increase in column height was found to decrease sorption efficiency and increase breakthrough time [102, 109]. An increase in initial concentration of sorbate enhanced sorption capacity and decreased breakthrough time [100, 109]. Increasing the influent flow rate also decreased the breakthrough time [109]. In a dynamic study on the sorption of phenols onto olive pomace, decreasing flow rate and particle size was found to improve sorption capacity [110].

### 3.8. Competition From Coions

One additional factor affecting biosorption in multicomponent systems is competition and interference between ions in the sorbate mixture. As a result, the reported individual batch uptake and breakthrough capacities of ions in single-component systems were lower compared to their counterparts in multicomponent systems [123]. However, high removal efficiency (up to 80–90%) was achieved in multicomponent systems of heavy metals [71, 103]. It was also suggested that competition is minimized at low ion concentrations [57, 122]. In general, Pb ions showed more competitiveness than Cd ions onto different adsorbents such as grape bagasse [79], olive mill waste [102], biomass from sunflower oil [112], and fruit waste macrofungi [130].

## 4. Nature and Mechanism of Biosorption

Food and pharmaceutical wastes contain organic compounds such as proteins, amino acids, polysaccharides, phenolics, and acids. These compounds have functional groups that bind to the sorbate cations. Groups include, but are not limited to, amines, hydroxyls, carbonyls, sulfonyls, thiols, and phosphates. Biosorption mechanisms include physical sorption by virtue of Van der Waals forces or by ion exchange electrostatic interactions, chemical sorption by chelation or complexation, and microprecipitation. Generally, a combination of these mechanisms is involved in biosorption [88, 132, 133].

There are several factors controlling sorption mechanisms, type of ligands or binding sites available on the sorbent; chemical structure and characteristics of the target ions/molecules, physicochemical conditions such as pH, ionic strength, and temperature. There are some general rules for metal binding particularly via complexation. Hard acids such as K^+^, Na^+^, Ca^2+^, and Mg^2+^ prefer to bind to oxygen ligands, whereas soft acids such as the precious metal ions of Ag, Au, Hg, and Cd preferentially bind covalently to the cell wall via ligands that contain nitrogen or sulfur [132, 134].

Sorption onto biomass can generally occur via one or more of the following mechanisms: rapid surface reaction between the sorbate and the active functional groups existing in the cell wall, intracellular accumulation, or precipitation/extracellular accumulation. Surface reaction could be either physical adsorption or chemisorption and is nonmetabolism dependent. Intracellular accumulation takes place when the sorbate migrates across the cell wall. It is a metabolism-dependent process that is influenced by adverse environmental conditions such as lack of nutrients and toxicity. It is also a function of the regular metabolic activities that change the microenvironment surrounding the cell, such as nutrient uptake, metabolic release, and respiration. In living biomass, biosorption is metabolism-dependent and occurs by sorbent uptake across the cell membrane. Therefore, it has its limitations regarding toxicity and maintaining nutrient levels. Biosorption via dead biomass does not suffer from these limitations and occurs on the cell wall where the polysaccharides and proteins have binding sites. However, lower binding capacities and higher desorption tendencies are often encountered [88, 135, 136].

To elucidate the underlying biosorption mechanism, the functional groups involved in biosorption were determined by Fourier transform infrared spectroscopy (FTIR) analysis (Table 2). Generically, sorption onto pectin-rich fruit wastes involved hydroxyl and carboxyl groups [94, 95, 101, 102]; whereas sorption onto olive oil wastes involved carboxylic and phenolic groups [102, 108, 111]. Biomass fungal wastes had additional amine groups as in* Aspergillus Fumigatus* and* Aspergillus awamori*. These groups were donated by the chitosan and chitin that are predominantly present in the fungal cell walls [122, 124, 125]. A two-step mechanism was suggested for sorption onto* Aspergillus awamori* where there is an initial adsorption step followed by a chromium reducing step from Cr (VI) to Cr (III). Heavy metal sorption onto okra food wastes and sugar bagasse wastes took place via a combined ion exchange/complexation mechanism where the positively charged metal interacted with the negatively charged wastes. The negative charge on the wastes was owed to the presence of lone pairs of the nitrogen and oxygen atoms that exist in the functional groups of cellulose, lignin, protein, and sugar [88, 89]. The main functional groups responsible for dye sorption onto pecan nut shells and mango seeds were the sulfonyl groups. Mechanism of sorption onto the former biosorbent depended on the number of sulfonic groups present on the dye; these groups interacted with the Ca compounds belonging to the pecan nut shells [131]. The mechanism of dye sorption onto the latter biosorbent entailed association with water molecules which linked the Victazol Orange 3R sulfonic groups to the syringyl groups of the lignin cellulose present in the mango seeds [99]. A variety of functional groups played important roles in the biosorption of Pb onto waste beer yeast. The extent of contribution of these groups was in the descending order: carboxylic, lipids, amines, and phosphates [82]. For the sorption of anionic dyes onto Cupuassu shells, a 3-step mechanism was proposed. It involved an initial rapid step for protonation of the Cupuassu shells functional groups. This was followed by dissociation of the dye agglomerates and their consequent dehydration, then finally electrostatic binding between the negatively charged dye and the positively charged biosorbent. Activated carbon from the pharmaceutical antibiotic waste contained primarily oxygen-containing functional groups such as hydroxyl and carbonyl groups which formed complexes with the mercury ions [126].

The change in pH during sorption could be indicative of the involved mechanism. For example, the decrease in pH during the sorption of heavy metals onto* Tolypocladium* sp. is a result of proton release probably due to electrostatic interaction between the positively charged metals and the negatively charged carboxylic groups on the adsorbent [107].

## 5. Equilibrium and Kinetic Modeling Studies

Biosorption equilibrium is governed by isotherm models that are well-known and established in literature.  Table 3 [82, 86, 87, 127, 131, 137] summarizes the different isotherm model equations involved in the present review along with their relevant parameters. For the kinetic modeling, the reported studies herein were found to follow either pseudo-first order or pseudo-second order or Elovich models; equations thereof are presented in Table 4 [94, 98, 131, 137].

Table 5 compiles a summary of the sorption parameters pertaining to the studies utilizing food and pharmaceutical waste biosorbents, as predicted by the different well-established models for sorption equilibrium. The table presents only results that were obtained by the best fitting model relevant to each study. In the majority of equilibrium studies, heavy metals and dyes were shown to follow Langmuir isotherm. This indicates single-site monolayer binding where the surface of sorbent is homogenous and all sites are equally favorable or nonfavorable from the energetic point of view. Few heavy metals followed Freundlich isotherm which assumes a heterogeneous biosorbent surface; examples are Cd (II) [123] and Pb (II) [82, 97, 105]. Cd was also shown to follow BET [120] and Sips [94], while Pb was found to follow Sips [102] and Dubinin Astakhov [103]. Several dyes such as Reactive Red (RR 194), Direct Blue 53, Acid Blue 25, and Reactive Black 5 followed Sips isotherm [87, 127, 131], whereas Reactive Red dye (RR 198) and phenols followed Freundlich isotherm [86, 110]. In general, it can be observed from the table that sorption capacities of pharmaceutical and fungal biomass wastes for heavy metals and dyes are higher than those of pectin-rich fruit wastes or olive oil wastes. Sorption in multicomponent systems was well described by either Langmuir as in case of simulated acid bath for wool (SABW) dye mixture [121] or extended Langmuir as in case of binary mixtures of heavy metals [105].

In most reported studies, the pseudo-second order model was found to be the most appropriate fitting model that describes biosorption of heavy metals and dyes onto food and pharmaceutical wastes (Table 6). This indicates that the mechanism is that of chemisorption. The model takes into account the three phases of the sorption process; surface reaction, film or external diffusion, and pore or intraparticle diffusion. Generally, time versus uptake trends revealed fast kinetics where almost 90% or more of the material was sorbed within a time range from few minutes to few hours and equilibrium was almost approached. In one instance when Cr (III) was adsorbed onto orange waste, reaching very close to complete equilibrium required a few days [102]. This could be owed to a diffusion-controlled sorption taking place onto the very porous pectin-rich biosorbent as was alluded to in Section 3.5.

## 6. Pretreatment and Regeneration/Recovery Options

Wastes from most fruit sources are pectin-rich biosorbents of potentially high metal binding abilities. Many studies that involved the use of such biosorbents have undertaken prior chemical pretreatment known as protonation. This process aims at removing excess cations such as Ca^2+^, Na^+^, or K^+^ from the biosorbent before carrying out biosorption experiments to reduce the competition of these elements with targeted heavy metals. Moreover, it leads to the creation of negative active sites on the biosorbent surface (at specific pH values) which leads to higher metal uptake capacity [92]. Some researchers utilized HNO_3_ with predetermined concentration for this chemical pretreatment step. A comparative study [101] was carried out on the performance of treated and untreated orange peel waste. Results showed higher Cd (II) uptake of 11.2 mg/g in case of chemically treated orange peel as compared to 6.94 mg/g in case of original peel. Similarly, [138] pectin waste was protonated by HNO_3_ before carrying out batch biosorption experiments for heavy metal removal. It was also suggested in a different study [99] that the use of HCl was successful in acidifying the mango seeds (AMS) in order to improve its removal efficiency to dyes. A faster adsorption process accompanied by increase in adsorption capacity was encountered in case of AMS relative to the original mango seeds (MS). According to them, the protonation using HCl increased the macropore structure of the original MS allowing higher amounts of dyes to be adsorbed.

H_2_O_2_ along with thermal treatment was used to treat wine processing sludge in order to remove organic matter before using the biosorbent for the removal of Cr (VI) [73]. This step led to the reduction of Cr (VI) to Cr (III) which reached a percentage of 2–18 at the end of the biosorption experiment. They considered pretreatment as a key factor for any future research to be performed on Cr removal using the same biosorbent. A comparison between chemically treated and untreated waste olive cake biomass during a study for Zn (II) removal from aqueous solutions was conducted [108]. This showed an increase in the removal efficiency of waste treated by NaOH and a reduction in the removal efficiency in case of H_2_SO_4_ treated waste.

A combined chemical and physical treatment was performed for baker's yeast waste used for Cd (II) and Pb (II) removal [139]. Thermal treatment (121°C), NaOH, and ethanol were applied to separate biomass samples and the resulted treated biomass was employed in batch biosorption experiments. The best metal uptake was reported for ethanol treated biomass whereas caustic and thermal. Caustic soda and ethanol were utilized for pretreating the* Penicillium oxalicum var. Armeniaca* biomass used for heavy metal removal [107]. The purpose was to remove proteins from the biomass sites via caustic treatment which increased biosorption capacity, whereas the opposite was true in case of acidic pretreatment.

Other different chemical treatments either acidic or caustic were employed by other workers in their biosorption studies. Zinc chloride and potassium hydroxide were employed as chemical activating agents for palm oil sludge in a study on the removal of Methylene Blue dye [111]. Results were comparable to commercial activated carbon but chemically treated sludge was recommended for better solution to sludge disposal problems. Simulation of chemical and physical pretreatment was employed in another study on the removal of phenols from industrial wastewaters using olive pomace solid wastes produced from different stages of olive mills [110]. Dried olive pomace denoted OP-1, solvent extracted using hexane and vapor olive pomace (OP-2), solvent extracted and incompletely combusted in boilers, and olive pomace (OP-3) were utilized. Results recommended the use of OP-3 as an effective biosorbent for phenol removal as the reported removal efficiencies exceeded 90%. The use of sugarcane bagasse modified by EDTA dianhydride for the removal of MB and GV dyes was investigated by other workers [78], and there was no sign of its effectiveness as a biosorbent compared to the untreated bagasse. It has also been reported in another study that the physically and chemically treated waste cider yeast was efficient in removing patulin [84]. Heating and chemical addition of either NaOH or ethanol was done during yeast preparation for batch experiments, while calcium alginate was used to form a gel bead (cell immobilization) with and without NaOH treatment for the column study. For batch results, the highest % removal obtained was 58.29% in case of caustic treatment and 44.41% for thermal or ethanol treatment. Column results showed substantial improvement in patulin removal by caustic treated immobilized yeast matrix (100% removal) as compared to untreated yeast (71.42%). Another cell immobilization was performed during a study conducted on the removal of heavy metals using waste biomass from beverage industry [75]. Comparison between the removal efficiencies of both immobilized (on Dowex resin and Chitin) and free biomasses indicated limited improvement in uptake performance. Another study used polyvinyl alcohol for biomass immobilization [122]. This process helped in improving biomass adsorption capacity and regeneration ability. A different study compared the performance of immobilized and suspended brewery waste yeast biomass versus fresh yeast (from baker's) in the removal of Cd (II) from aqueous solutions [80]. An excellent removal efficiency of 99.83% was reported in case of suspended form of brewery waste (SBW) which confirmed its promising potential as an effective low-cost adsorbent.

To test the effect of different functional groups on the removal of some heavy metals (Cd^2+^, Zn^2+^, and Cr^3+^), orange waste biomass was chemically modified by specific reagents [95]. It was suggested that ester treatment was not recommended as a prior modification because it decreased biosorption capacity. Five different chemical reagents were applied to chemically treat the waste beer yeast used in biosorption of lead from electroplating effluents [82]. Lead removal efficiencies were reduced after using all five pretreatment methods with drastic effects shown in case of ethanol and HCl treatment. Similar results were reported by the same workers in their study on Cr removal [74]. Their results showed reduction in removal efficiency using all types of treating agents relative to the original biosorbent and they owed this to the creation of modified functional groups.

Some researchers used chemical and physical methods for the preparation of activated carbons produced from different food processing wastes. Four studies were reported for the removal of dyes or heavy metals using AC prepared from exhausted olive waste cake [113], AC prepared from oil palm empty fruit bunch [114], AC prepared from tea waste [115], and AC prepared from sago waste [116]. Chemical activation was performed using sulfuric or phosphoric acids or potassium hydroxide and this was followed by heating to relatively high temperatures or microwave assistant heating as an essential step in all four studies. Reported results showed an outstanding performance for AC from tea industrial waste in Cr (III) removal with an efficiency that almost reached 100%.

As for pharmaceutical wastes,* Tolypocladium* sp. was treated with methanol to improve its sorption capacity for Hg (II) [107]. Modification of the amino groups on the waste mycelium of* Aspergillus awamori* via treatment with formaldehyde, acetic anhydride, or sodium iodoacetate did not improve its removal efficiency for Cr (VI) [124]. The treatment of the same biosorbent with sodium hydroxide and dimethyl sulfoxide increased Cu (II) uptake [125]. Among different pretreatments of* Corynebacterium glutamicum* with HCl, H_2_SO_4_, HNO_3_, NaOH, Na_2_CO_3_, NaCl, and CaCl_2_, the maximum improvement in biosorption capacity for Reactive Black 5 dye was achieved with 0.1 M HNO_3_. This was attributed to the enhancement of positively charged cell surfaces that attract the negatively charged dyes [127].

Few researchers were interested in desorption processes for either regeneration of the biosorbent for reuse and/or for recovery of the sorbate material. Desorption can be performed by adding acids, bases, inorganic salts, or solvents [129] for metal recovery. This step usually follows the adsorption step and metal recovery rate or metal uptake is calculated to test the effectiveness of the reagent used in desorption. HCl was the most used eluent for the majority of the reported studies [100, 101, 103–105, 116, 122, 123, 129]. The most promising reported results showed that olive mill waste (OMW) maintained its adsorptive capacity for Cd (II) and Pb (II) after ten adsorption-desorption cycles [103]. HCl elution was followed by neutralization with Na_2_HCO_3_ [101]. Other workers revealed that exhausted modified orange peel was able to adsorb Pb (II) up to 91.5% after the 4th cycle. It was concluded in another study that desiccated coconut waste can be used multiple times for Hg sorption and can be regenerated easily using HCl [100]. It was also found in other batch experiments that 41.7% recovery of Fennel biomass could be attained after the 5th cycle using HCl as an eluent [123]. In addition, the relevant column study was very successful and resulted in 87.8% of Cd (II) being eluted in a single-component system and almost 100% in a multicomponent system. Results of using HCl as an eluting agent showed that after the 4th cycle, 65–70% of initial Cd (II) can be retained onto* Aspergillus Fumigatus*, but the biosorbent deteriorated after the 5th cycle [122].

In comparing HCl with other desorping agents, higher Pb (II) desorption rate using HCl as compared to EDTA was reported [26]. However, EDTA manifested better performance than both HCl and CaCl_2_ (equal desorption effect) in case of Cu (II) desorption but had the disadvantage of damaging the biosorbent sites [104]. Furthermore, the regenerated waste was able to remove 40% of Cu (II). Different results revealed that Pb (II) was better recovered by HNO_3_ (desorption rate of 76.6%) while Cd (II) was better recovered by HCl (desorption rate of 62.5%) [129]. The desorping agent, HNO_3_, was utilized in another study where the results showed that the regenerated biomass was successfully capable of desorping Cd (II) even after 5 consecutive cycles [120]. The comparison between the use of HNO_3_ and double deionized water (DDW) in desorbing Ni (II) showed that DDW was very poor compared to HNO_3_ [76]. The same acid was also used along with another two desorbing agents, NaNO_3_ and Ca(NO_3_)_2_, to regenerate peels and it was shown that regenerated peels have the same efficiency as the original peels [140]. About 90–100% of Cd (II) was recovered from the peels in 120 min or less. Additionally, HNO_3_ gave superior removal efficiency of 90% in only 50 min without damaging the peels.

## 7. Concluding Remarks

Industrial food processing and pharmaceutical wastes are promising biosorbents for treatment of wastewater effluents. They contain functional groups such as hydroxyl, carboxyl, and amine that allow them to interact with metal ions and dye pollutants via physical and/or chemical sorption. Sorption equilibrium in most of the previous studies was best described by Langmuir isotherm suggesting single-site binding. Sorption kinetics was generally fast and it predominantly followed the pseudo-second order model indicating a chemisorption mechanism. Surface reaction as well as film and pore diffusion processes were considered in the model.

Biosorption is influenced by the physical and chemical properties of the sorbent as well as various operating conditions. Numerous workers studied the effect of these parameters in batch systems. However, very few studies were conducted in continuous column systems. The latter is of paramount importance in scaled-up applications. Furthermore, most workers employed synthetic aqueous solutions rather that real wastewater effluents where competition and interference between ions in the mixture could significantly affect biosorption performance. One parameter that was overlooked is the physical, mechanical, and chemical stability of the sorbent. Mechanical strength of the biosorbent and its resistance to chemicals and microbial degradation are crucial parameters that ensure reproducibility of biosorbent, particularly in continuous operations where the biosorbent is regenerated and reused many times. Maintaining reproducibility for many subsequent cycles has both environmental and economic merits.

Desorption studies are relatively fewer compared to removal studies. The former is particularly important for both biosorbent regeneration and sorbate recovery if of value. Disposing of, landfilling and incineration could be alternatives to discarding the used biosorbent rather than regenerating it. However issues with cost and leaching of toxic compounds in the soil and ground water make them sometimes unfavorable options. Under very strong binding conditions, where the equilibrium binding constant is high, leaching and metal release are minimized.

Biosorbent performance could be enhanced by chemical, thermal, or chemical/thermal pretreatment and/or immobilization. Pretreatment could be performed to remove undesired organic compounds, proteins, or competing ions from the biosorbent and hence improve biosorption capacity and efficiency. In other cases, pretreatment is undertaken to add new functional groups to the biosorbent that can possibly enhance biosorption. However in some cases, pretreatment gave adverse effects. Prior characterization studies on the biosorbent may help in selecting the suitable treatment option.

Biosorption utilizing industrial food processing and pharmaceutical wastes could provide a cost-effective ecofriendly viable means of treating wastewater effluents, while making good use of waste materials. However, more work should focus on scaling up the proposed biosorption processes and studying their technoeconomic feasibility. Research should also be extended to using these biosorbents for treatment of different classes of contaminants such as phenolic compounds and mycotoxins.

## Figures and Tables

**Table 1:** Summary of the different industrial food processing and pharmaceutical waste biosorbents; their sources, applications, and the relevant biosorption parameters.

Type of biosorbent	Source of biosorbent	Feed solution	Sorbate	pH	Contact time, min	Temperature, °C	Initial concentration of sorbate, mg/L	Mode of operation	Maximum %removal	Biosorbent dose	Reference
Spent brewery grains (SBG)	Mohan breweries and distilleries Limited, Chennai, India	Synthetic dye solution	AG25 acid dye of commercial name Alizarin Cyanin Green G	3.0	75	30	90	Batch	98	0.2	[70]

Tea industry waste	Local tea factory in China	Aqueous synthetic solution	Cd(II)	7.0	180	25	20	Batch	90	5	[72]

Exhausted coffee waste	Soluble coffee manufacturer, Catalonia, Spain	Aqueous synthetic solution	Cr(VI) Cu(II) Ni(II)	3.0	8640	25	1000	Batch	—	6.67	[77]

Sugarcane bagasse waste	From local alcohol and sugar	Aqueous synthetic solution	Methylene Blue (MB)	7.0	600	25	200	Batch	—	0.2	[78]
	Industries, City of Ouro Preto, Minas Gerais, Brazil		Gentian Violet		900		300				

Wine processing waste sludge (WPWS)	Ilan Wine-Processing Company, Ilan, Taiwan	Aqueous synthetic solution	Cr(III, IV)	2.0	240	30	100	Batch	36	10	[73]

Wine processing waste sludge (WPWS)	Ilan Wine-Processing Co., Ilan, Taiwan	Aqueous synthetic solution	Ni(II)	5.5	120	50	30	Batch	75	12	[76]

Grape bagasse waste residue	Wine production process, Styria region, Austria	Effluent from research laboratory	Cd(II) Pb(II)	7.0 3.0	45	25	100	Batch	—	0.67	[79]

Waste beer yeast	Aoke Beer Company in Zhengzhou, Henan province, China	Aqueous synthetic solution	Cu(II) Pb(II)	5.0	30	20	9.14 32.23	Batch	—	—	[69]

Waste beer yeast *Saccharomyces cerevisiae *	Beer fermentation industry, brewery located near Chennai, India	Electroplating effluents	Cr(VI)	5.0	120	—	—	Batch	—	0.02	[74]

Suspended brewery yeast waste biomass (SBW)	Brewery waste biomass collected from CIUC brewery, Miercurea-Ciuc, Romania	Synthetic aqueous solution	Cd(II)	5.5	40	50	6	Batch	99.83	9.78	[80]

Beer brewery diatomite waste (SDE)	Shan-Hua factory, Tobacco and Liquor Co., TainanTaiwan	Synthetic aqueous solution Industrial wastewater from local factory	Methylene Blue (MB) basic dye	7.0	1440	25	2.5	Batch	—	0.25	[81]

Spent waste beer yeast *Saccharomyces cerevisiae *	Fermentor at a brewery, Chennai, India	Battery manufacturing industrial effluent	Pb(II)	5.0	120	30	100	Batch	—	—	[82]

Fresh malted sorghum mash waste	local malted sorghum beer (pito) brewer at Navrongo, Ghana	Synthetic aqueous solution	Methylene Blue (MB) basic dye	7.0	18	33 ± 1	50	Batch	>90	4	[83]

Waste biomass from sugarcane aguardente	Brazilian alcoholic beverage production, (Lapinha, Bocaiana, Germana and Taboroa) State of Minas Gerais, Brazil	Stainless steel effluent	Cr(VI) Fe(III) Ni(II)	4.0	180	25	50 660 20	Batch	70 50 20	1	[75]

Waste biomass Cachaça Brazilian alcoholic beverage	the stillage generated by a liquor distillery (Germana), Minas Gerais, Brazil	Stainless steel industrial effluent from Acesita Co., Brazil	Fe(III) Ni(II) Cr(VI)	4.0	180	25	7.8 2.7 600	Batch	94 57 25	2	[71]

*Rhizopus oligosporus* biomass	Food processing wastewaters	Aqueous synthetic solution	Cu(II)	5.0	120	30	100	Batch	70	1	[85]

Waste biomass of *Phaseolus vulgaris L. *	A residual biomass of a canned food factory in Bartin, Turkey	Aqueous synthetic solution	Pb(II)	5.0	20	20	100	Batch	92	4	[84]

Waste biomass of *Phaseolus vulgaris L. *	A residual biomass of a local canned food plant, Turkey	Aqueous synthetic solution	Textile Reactive Red dye (RR 198)	2.0	20	20	100–300	Batch	99.3	1.6	[86]

Cupuassu shell, *Theobroma grandiflorum*, (CS)	Food residue from jelly industry, Belém-PA, Brazil	Aqueous synthetic solution	Reactive Red dye (RR 194)	2.0	480	25	50	Batch	—	2.5	[87]
			Direct Blue 53		1080						

Okra food industrial waste	Food waste from food canning processes	Aqueous synthetic solution	Cd(II) Fe(II) Zn(II)	—	90	—	20	Batch	96.4 93.8 79.8	1	[88]

Sugar industrial waste (bagasse waste)	Obtained from food canning processes	Aqueous synthetic solution	Cd(II) Fe(II)	—	90	—	20	Batch	96.4 93.8	1	[89]

Pineapple peel, an agricultural effluent	Food can processing industries	Aqueous synthetic solution	Methylene Blue (MB) cationic dye	6.0	400	30	300	Batch	47	1.5	[91]

Waste baker's yeast biomass	Pakmaya Yeast Company, Izmir, Turkey	Aqueous synthetic solution	Cd(II) Pb(II)	6.0 5.0	180	30	25	Batch	60 70	1	[131]

Desiccated coconut waste sorbent (DCWS)	By-product of Coconut Milk Processing	Aqueous synthetic solution	Hg(II)	7.4	2880 400	30	50 100	Batch Column	—	1 *F* = 4 mL/min	[100]

Pecan nut shells (*C. illinoinensis*) biomass	Biomass from food factories, Nuevo Leon, Mexico	Aqueous synthetic solution	Acid Blue 74 (AB74)	6.5	500	30	100	Batch Column	—	10 *F* = 3 mL/min	[132]
			Reactive Blue 4 (RB4)		1000						
			Acid Blue 25 (AB25)		500						

Orange peel	Solid waste from local fruit juice industries, Egypt	Aqueous synthetic solution	Pb(II) Cu(II) Cd(II)	5.0	30	25	20	Batch	99.5 89.57 81.03	4	[101]

Orange (*Citrus sinensis*) waste	Agrumexport, S.L., an orange juice manufacturing company located in Murcia, Spain	Aqueous synthetic solution	Cr(III)	4.0 4.0	4320 1523	25 25	100 20	Batch Column	81 57.5	4	[102]

Pectin-rich fruit wastes	Residues from fruit juice and wine production, from a citrus-juice producer (Sunkist), USA	Aqueous synthetic solution	Cd(II)	5.0	50	—	60	Batch	46	2	[133]

Orange waste	From orange juice industry, Spain	Aqueous synthetic solution	Cd(II)	6.0	60	25	100	Batch	98	4	[94]

Orange waste	From orange juice industry, Spain	Aqueous synthetic solution	Cd(II) Zn(II) Cr(III)	4.0	180 180 4320	20	15 15 15	Batch	86 90 95	4	[95]

Peach and Apricot stones	Solid wastes of juice and jam industries, Egypt	Aqueous synthetic solution	Pb(II)	7.0	180 300	—	54.65	Batch	97.64 95.3	10	[97]

*Mangifera indica* (mango) seed kernel particles	Local juice manufacturing industry	Aqueous synthetic solution	Methylene Blue (MB) cationic dye	8.0	120	30	100	Batch	96.17	0.67	[98]

Mango seeds (MS) *Mangifera indica L. *	Juice producer, Ubá-MG, Brazil	Aqueous synthetic solution	Victazol Orange 3R dye (VO-3R)	2.0	360	25	40	Batch	—	2.5	[99]

Waste cider yeast biomass	Fermentation Lab at the College of Food Science and Engineering of Northwest A & F University (Yangling, China)	Apple juice solution	Patulin (PAT)	4.5	2160	25	0.1 0.2	Batch Column	58.29	5 *F* = 2 mL/min	[117]

Dairy sludge	Dairy plant, France	Aqueous synthetic solution	Pb(II) Cd(II)	5.0	500	20	200 100	Batch	>90	0.5–4.0	[129]

The waste pomace of olive oil factory (WPOOF)	Turkish Prina, Aegean region, Manisa, Turkey	Aqueous synthetic solution	Cr(VI)	2.0	120	60	50 100	Batch Column	100 21.74	5 *F* = 5 mL/min	[101]

Solid waste from olive oil production	The OS and OMS wastes were provided by the “Cooperativa Nuestra Se^~^nora desl Castillo” extraction plant located in Vilches, in the province of Jaen (Spain)	Aqueous synthetic solution	Pb(II)	5.0	120	25	—	Batch	—	10	[102]

Olive mill waste (OMW) two-phase decanter	Mixture of pulp and olive stones from the crushing of olives to obtain the oil, UK	Aqueous synthetic solution	Pb(II) Cd(II) Cu(II) Hg(II) Fe(II)	7.0	30	20	10	Batch	80 75	10	[103]

Olive mill residues (OMR)	Solid residues of oil production, provided by an olive mill in Abruzzo, Italy.	Aqueous synthetic solution	Cu(II)	5.5 4-5	150–1440	Room temperature	40 40	Batch Column	60% —	10 80	[104]

Palm oil mill effluent (POME) sludge	Waste sludge from palm oil mill, Felda Taib Andak, Johor, Malaysia	Aqueous synthetic solution	Methylene Blue (MB) cationic dye	7.5	4320	27	100	Batch	—	2	[111]

Sunflower oil Waste biomass	Biomass obtained from a sunflower oil production Facility, Seville, Spain	Aqueous synthetic solution (mixed solution)	Pb(II) Ni(II) Zn(II) Cu(II)	4.0	—	—	10	Column	—	*F* = 2 L/h	[112]

Crushed olive stone wastes	Supplied by an olive oil Producer, Cordoba, Spain	Aqueous synthetic solution	Pb(II) Ni(II) Cu(II) Cd(II)	5.5	60	20	18.86 4.20 4.35 4.80	Batch	79 70 81 95	13.3	[105]

Waste olive cake (OC)	Supplied by “ProBeira” an olive oil producer, Envendos Portugal	Aqueous synthetic solution	Zn(II)	6.0-7.0	120	25	10	Batch	93	1	[108]

Olive stones	From the orujo oil extraction plant ‘‘Orujera Ubetense, Sociedad Cooperativa Andaluza,” Jaen, Spain	Aqueous synthetic Solution	Cd(II)	11	360	40	10	Batch	90	0.01	[106]

Olive pomace	Supplied by an Italian olive oil production plant	Aqueous synthetic Solution	Cu(II) Cd(II) Pb(II)	5.0	60	—	—	Batch	—	10	[107]

Olive pomace	Supplied by one of the olive oil production Plants, Jordan	Aqueous synthetic Solution	Methylene Blue (MB) dye	—	240	25	10 40	Batch Column	80 62.25	2 *F* = 20 mL/min	[109]

Olive pomace OP-1 OP-2 OP-3	Solid by-products of olive oil processing mills, the island of Lesvos, Greece.	Oil mill waste water (OMWW)	Phenol	10.0	120	20	50 500	Batch Column	>90 90	10 *F* = 1 mL/min	[110]

Activated carbon derived from exhausted olive waste cake	Olive waste cake from oil factory “Agrozitex” Sfax, Tunisia	Synthetic aqueous solution Industrial wastewater from local factory	Lanaset Grey G	6.0	3000	25	150	Batch	93	1.67	[113]

Activated carbon derived from empty fruit bunch (EFB)	Industrial waste from united palm oil mill, Nibong, Tebal, Malaysia	Synthetic aqueous solution	Methylene Blue dye (MB)	12	—	30	200	Batch	—	1	[114]

Activated carbon from tea industry waste (TIWAC)	Tea waste from tea processing plant, Black Sea region, Trabzon, Turkey	Real water samples	Cr(VI) Cr(III)	6.0	30	—	0.2	Batch	0 95–100	2	[115]

Activated carbon from sago waste	Sago waste is collected from sago industry, Salem district, Tamilnadu, India	Synthetic aqueous solution Industrial wastewater from radiator industry	Pb(II)	3.5 ± 0.3	180	27	10	Batch	87.34	2	[116]

*Phaseolus vulgaris L. *	Canned food factory	Aqueous synthetic solution	Acid Red 57 dye	2.0	20	20	150	Batch	—	1.6	[91]
			Textile wastewater				1 (spiked wastewater sample)		97.68		

Industrial fungi *Penicillium oxalicum var. Armeniaca *	Ascolor Biotec (Pardubice, Czech Republic) and Ivax Pharmaceuticals (Opava, Czech Republic)	Aqueous synthetic solution	Pb(II) Hg(II) Cd(II)	5.0	—	20	10 50 50	Batch	Up to 85 for Hg	0.3	[118]

Fruit waste macrofungi *Flammulina velutipes *	Mushroom processing factory	Aqueous synthetic solution	Cd(II) Pb(II)	6.0	60	25	10	Batch	75 70	18	[130]

*Tolypocladium* sp.	Czech Industrial Partners	Aqueous synthetic solution	Cd(II) Pb(II) Hg(II)	5.0	20	—	50	Batch	—	0.3	[118]

Antibiotic waste *P*. *mutilus *	SAIDAL antibiotic production complex at Medea (Algeria)	Aqueous synthetic solution	Basic Blue 41, cationic dye	8.0-9.0	60	30	50	Batch	75	0.5	[119]

Industrial waste of *Clitopilus scyphoides *(*Pleurotus mutilus*) fungal biomass	Antibiotic production plant, SAIDAL antibiotic production complex in Médéa (Algeria)	Aqueous synthetic solution	Cd(II)	5.0	15	20	200	Batch	41	1	[120]

Fungal waste biomass	Pharmaceutical companies, Italy	Textile wastewater effluents	Dye mixtures	3.0	30	25	60–5000	Batch	90	16.7	[121]

Nonliving biomass *Aspergillus fumigatus *	Fermentation industry, Artemis Pharmaceuticals Limited, HAD, Jeedimetla, Hyderabad, India	Aqueous synthetic solution	perse mixture Cu(II) Cd(II) Co(II) Ni(II)	7.0	60	—	4.8 2.9 4.8 2.7 2.8	Batch	70 90	20 8	[122]

Nonliving biomass *Aspergillus awamori *	Industrial complex enzyme preparation	Aqueous synthetic solution	Cr(VI)	2.0	40	1152	25	Batch	87	1	[124]

Nonliving biomass *Aspergillus awamori *	Industrial complex enzyme preparation	Aqueous synthetic solution	Cu(II)	5.0	20	180	100	Batch	—	1	[125]

*Corynebacterium* * glutamicum *	Fermentation industry (BASF-Korea)	Aqueous synthetic solution	Reactive Black 5 (RB5)	1.0	35	500	500	Batch	—	2.5	[127]

Activated carbon from antibiotic waste	Industrial antibiotic production	Aqueous synthetic solution	Hg(II)	5.5	—	30	40	Batch	—	0.2	[126]

Fennel biomass (*Foeniculum vulgare*)	Medical herb, local Unani medicine manufacturing unit at Aligarh, India	Aqueous synthetic solution	Cd(II) Mixture Cd(II) Ni(II) Zn(II) Cu(II)	4.3	50 50 each	50	100	Batch Column	92 97	10 *F* = 1 mL/min	[123]

**Table 2:** Suggested biosorption mechanisms based on interacting functional groups.

Biosorbent	Sorbate	Functional group	Mechanism	Reference
Orange peel	Pb(II) Cu(II) Cd(II)	Carboxylic	IEX/H-bonding	[101]

Orange waste	Cr(III)	Carboxyl/hydroxyl	Chemisorption	[102]

Orange waste	Cd(II)	Carboxyl/hydroxyl	—	[94]

Orange waste	Cd(II) Zn(II) Cr(III)	Mainly carboxyl	—	[95]

Desiccated coconut	Hg(II)	Hydroxyl/carboxyl/amine	Chelation	[100]

Pecan nut shells (*C. illinoinensis*) biomass	Acid Blue Reactive Blue Acid Blue	Sulfonyl	—	[132]

Cupuassu shell, *Theobroma grandiflorum*, (CS)	Reactive red dye Direct blue	Hydroxyl/carboxylic	IEX	[87]

Mango seeds (MS) *Mangifera indica L*.	Victazol orange	Sulfonyl	—	[99]

Okra food industrial waste	Cd(II) Fe(II) Zn((II)	Hydroxyl/carbonyl/amide	IEX/complexation	[88]

Sugar industrial waste (bagasse waste)	Cd(II) Fe(II)	Hydroxyl/carbonyl/amide	IEX/complexation	[89]

Pineapple peel	MB dye	Hydroxyl/carboxyl/amine	—	[91]

Olive pomace	Pb(II) Cu(II) Cd(II)	Carboxylic/phenolic	Surface complexation	[108]

Olive mill stone	Pb(II)	Carboxylic	IEX	[102]

Palm oil mill effluent (POME) sludge	Methylene Blue	Carboxylic	—	[111]

Wine processing sludge	Ni(II)	Amino/carboxyl	Physical adsorption/chemical complexation	[76]

Spent waste beer yeast *Saccharomyces cerevisiae *	Pb(II)	Amine/carboxylic/ phosphates/sulfhydryl	IEX/complexation	[82]

Grape bagasse waste residue	Cd(II) Pb(II)	Carbonyl/hydroxyl	—	[79]

*Phaseolus vulgaris* biomass	Pb(II)	Amino/hydroxyl	—	[84]

Activated carbon from antibiotic waste	Hg(II)	Hydroxyl/carbonyl	Complexation	[126]

Nonliving biomass *Aspergillus awamori *	Cr(VI)	Amine	—	[124]

Nonliving biomass *Aspergillus Fumigatus *	Cd (II) *perse* mixture Cu(II) Cd(II) Co(II) Ni(II)	Hydroxyl/amine	Complexation	[122]

Fennel biomass (*Foeniculum vulgare*)	Cd(II)	Carboxylic/phenolic	IEX∗	[123]

*IEX: ion exchange adsorption.

**Table 3:** Main adsorption isotherm models involved in the present study.

Adsorption isotherm model	Model parameters
Freundlich *q* = *K*_*F*_C^1/*n*^ Linear form $\log q=\log K_{F}+\frac{1}{n}\log C$	*K*_*F*_: Freundlich isotherm constant that indicates adsorption capacity (mg/g) (L/mg)^*n*^ *n*: measure of adsorption intensity or surface heterogeneity (dimensionless) *q*: amount adsorbed at equilibrium, mg/g *C*: adsorbate concentration at equilibrium, mg/L

Langmuir $q=\frac{{q_{m}K}_{L}C}{1+K_{L}C}\;$ Linear form $\frac{1}{q}=\frac{1}{q_{m}K_{L}C}+\frac{1}{q_{m}}\;\;$	*q*_*m*_: maximum binding capacity, mg/g *K*_*L*_: Langmuir binding (adsorption) constant, L/mg

Sips $q=\frac{q_{m}{(K}_{s}{C)}^{1/n}}{1+{(K}_{s}{C)}^{1/n}}\;$ $q=\frac{k_{s}C^{\beta^{s}}}{1+a_{s}C^{\beta_{s}}}\;\;$	*K*_*s*_: Sips binding (adsorption) constant, (L/mg)^*n*^ *n*: dimensionless exponent constant *β*_*s*_ = Sips sorption exponent *a*_*s*_ = Sips sorption constant (L/mg)

Dubinin-Astakhov $q=q_{m}{\exp\left(-\frac{RT\ln\left(C_{s}/C\right)}{E}\right)}^{n}\;\;\;\;$	*E*: mean free energy of adsorption, KJ/mol *n*: Dubinin-Astakhov dimensionless exponent *T*: temperature, K *R*: universal gas constant, KJ/mol*·*K

BET $q=\frac{q_{m}C_{\text{BET}}C}{\left(C_{s}-C\right)[1+\left(C_{\text{BET}}-1\right)\left(C/C_{s}\right)]}\;$ Linear form $\frac{C}{q\left(C_{s}-C\right)}=\frac{1}{q_{m}C_{\text{BET}}}+\frac{\left(C_{\text{BET}}-1\right)}{q_{m}C_{\text{BET}}}\cdot \frac{C}{C_{s}}\;\;$ Alternative form $q=\frac{q_{m}b_{s}C}{\left(1-b_{L}C)(1-b_{L}C+b_{s}C\right)}$	*C*_BET_: BET isotherm constant which indicates energy of surface interaction, L/mg *C*_*s*_: saturation concentration of adsorbate, mg/L *b*_*s*_: isotherm constant for BET adsorption in the first layer, L/mg *b*_*L*_: isotherm constant for BET adsorption in upper layers, mg/L

**Table 4:** Equations of kinetic models involved in the current study.

Kinetic Model	Model Parameters
Pseudo-first order $\frac{dq_{t}}{dt}=k_{1}\left(q-q_{t}\right)$ Linear Form $\log\left(q-q_{t}\right)=\log q-\frac{k_{1}}{2.303}t$	*k*_1_: rate constant of pseudo-first order model, min^−1^ *q*: amount adsorbed at equilibrium, mg/g *q*_*t*_: amount adsorbed at time *t*, mg/g

Pseudo-second order $\frac{dq_{t}}{dt}=k_{2}{\left(q-q_{t}\right)}^{2}$ Linear Form $\frac{t}{q_{t}}=\frac{1}{k_{2}q^{2}}+\frac{1}{q}t$	*k*_2_: rate constant of pseudo-second order model, g/mg*·*min

Elovich $q_{t}=\frac{1}{\beta }\ln\left(\alpha \cdot \beta \right)+\frac{1}{\beta }\ln t$	*α*: initial adsorption rate, mmol/g*·*min *β*: Elovich constant, related to extent of surface coverage and activation energy, g/mmol

**Table 5:** Equilibrium parameters as predicted by the well-established sorption models.

Biosorbent	Target ion/compound	Equilibrium model	Maximum sorption capacity (mg/g)	Sorption constant∗	pH/temperature (°C)	Reference
Local dairy sludge	Pb(II) Cd(II)	Langmuir	178.6 69.90	0.03 0.05	5/40	[129]

Baker's yeast biomass	Cd(II) Pb(II)	Langmuir	31.75 60.24	0.092 0.066	6.0/30 5.0/30	[131]

Cider yeast	Patulin	Langmuir	0.0082	0.064	4.5/25	[117]

Beer yeast	Cu(II) Pb(II)	Langmuir	0.66 2.27	0.314 0.259	5.0/20	[69]

Spent waste beer yeast *Saccharomyces cerevisiae *	Pb(II)	Freundlich	—	*K*_*f*_ = 0.515 *n* = 0.842	5.0/30	[82]

Spent brewery grains (SBG)	AG25 dye	Langmuir	212.76	0.036	3.0/30	[70]

Wine processing sludge	Ni(II)	Langmuir	3.91	0.113	5.5/50	[76]

Antibiotic waste *P*. *mutilus *	Cu(II)	Langmuir	106.38	0.007	—	[119]

Antibiotic waste *P*. *mutilus *	Basic Blue 41	Langmuir Freundlich	111.00	0.097 *K*_*f*_ = 24.1 *n* = 2.89	(8.0-9.0)/30	[119]

*Phaseolus vulgaris L. *	Acid Red 57 dye	Langmuir	215.13	—	2.0/20	[91]

*Phaseolus vulgaris L. *	Reactive Red 198	Freundlich		*K*_*f*_ = 1.99 *n* = 10.037		[86]

Fruit waste macrofungi *Flammulina velutipes *	Cd(II) Pb(II)	Langmuir	8.43 18.35	—	6.0/25	[130]

Industrial fungi *Penicillium oxalicum var. Armeniaca *	Cd(II) Pb(II) Hg(II)	Langmuir	35.90 47.40 269.3	0.05 1.01 0.07	5.0/20	[107]

Industrial fungi *Tolypocladium* sp.	Cd(II) Pb(II) Hg(II)	Langmuir	11.90 28.40 161.0	1.03 0.61 0.50	5.0/20	[107]

industrial waste of *Clitopilus scyphoides* *(Pleurotus mutilus)* fungal biomass	Cd(II)	BET	45.3	16 *b*_*s*_ = 0.03 *b*_*L*_ = 0.00	5.0/20	[120]

Fungal waste biomass	Simulated acid bath for wool (SABW) dye	Langmuir	289.5	0.0114	3.0/25	[121]

biomass of *Phaseolus vulgaris L*.	Pb(II)	Langmuir	19.93	0.498	5.0/50	[84]

Fennel biomass (Foeniculum vulgare)	Cd(II)	Langmuir Freundlich	26.59	0.080 *K*_*f*_ = 3.16 *n* = 2.29	4.3/50	[123]

Nonliving biomass *Aspergillus awamori *	Cu(II)	Langmuir	35.97	0.136	5.0/20	[125]

*Corynebacterium * *glutamicum *	Reactive Black 5 RB5	Langmuir Sips	419	0.042 *k*_*s*_ = 108 *a*_*s*_ = 0.171	1.0/35	[127]

*Rhizopus oligosporus* biomass	Cu(II)	Langmuir	79.37	0.282	5.0/30	[85]

Pectin-rich fruit wastes (lemon peels)	Cd(II)	Langmuir	22.32	0.015	5.0/—	[133]

Orange waste	Cd(II)	Sips	20.64	0.038 (*n* = 1.21)	6.0/25	[94]

Orange waste	Cd(II) Zn(II) Cr(III)	Langmuir	17.66 14.61 22.50	0.004 0.067 0.372	4.0/20	[95]

Orange (*Citrus sinensis*)	Cr(III)	Langmuir	36.48	0.403	5.0/25	[102]

Pineapple peel, an agricultural effluent	Methylene Blue (MB) cationic dye	Langmuir	97.09	0.074	6.0/30	[91]

Peach stones Apricot stones	Pb(II)	Freundlich	—	*K*_*f*_ = 0.64 (*n* = 3.57) *K*_*f*_ = 0.636 (*n* = 3.54)	7.0/—	[97]

*Mangifera indica* (mango) seed kernel particles	Methylene Blue (MB) cationic dye	Langmuir	153.846	0.8227	8.0/50	[98]

Desiccated coconut	Hg(II)	Langmuir	500.00	—	7.4/30	[100]

Pecan nut shells (*C. illinoinensis*) biomass	Acid Blue 74 (AB74)	Langmuir	4.851	0.001	6.5/30	[132]
	Reactive Blue 4 (RB4)	Langmuir	13.410	0.001		
	Acid Blue 25 (AB25)	Sips	7.576	*K*_*s*_ = 0.0014 (*n* = 0.98)		

Crushed olive stone wastes	Pb(II) Ni(II) Cu(II) Cd(II)	Freundlich	—	—	5.5/20	[105]
	Binary mixtures	Extended Langmuir				

Olive pomace	Cu(II) Cd(II) Pb(II)	Langmuir	1.94 2.98 6.23	0.138 0.046 1.829	5.0/60	[108]

Olive pomace	Phenols	Freudlich	—	*K*_*f*_ = 0.267 *n* = 1.75	10.0/20	[110]

Pomace from olive oil	Cr(IV)	Langmuir	18.69	0.055	2.0/60	[101]

olive mill residues (OMR)	Cu(II)	Langmuir	13.50	0.080	5.0/23	[104]

Solid olive stone	Pb(II)	Sips	6.57	*K*_*s*_ = 0.057	5.0/25	[102]

Olive oil mill	Pb(II)	Dubinin-Astakhov	23.69		5.0/25	[103]

Palm oil mill effluent (POME) sludge	Methylene Blue (MB) cationic dye	Langmuir	23.50	0.208	7.6/27	[111]

Sugarcane bagasse waste	Methylene Blue (MB)	Langmuir	202.43	0.031	8.0/25	[78]
	Gentian Violet (GV)		327.83	0.047		

Fresh malted sorghum mash waste	Methylene Blue (MB) basic dye	Langmuir	384.6	0.011	7.0/53	[83]

Cupuassu shell, T*heobroma grandiflorum*, (CS)	Reactive Red dye (RR 194)	Sips	64.1	*K*_*s*_ = 0.214 (*n* = 0.89)	2.0/25	[87]
	Direct Blue 53		37.5	*K*_*s*_ = 1.560 (*n* = 0.55)		

Activated carbon derived from exhausted olive waste cake	Lanaset Grey G	Langmuir	108.70	0.031	6.0/25	[113]

Activated carbon derived from empty fruit bunch (EFB)	Methylene Blue dye (MB)	Langmuir	344.83	0.060	—/30	[114]

Activated carbon from sago waste	Pb(II)	Langmuir	14.35	0.095	3.5/27	[116]

*Units depend on the fitting isotherm model and are indicated in Table 3.

**Table 6:** Kinetic parameters as predicted by the well-established sorption models.

Biosorbent	Target ion/compound	Kinetic model	*q* (mg/g)	Rate constant∗	pH/temperature (°C)/time (min)	C_o_ (mg/L)	Reference
Local dairy sludge	Pb(II) Cd(II)	Pseudo-second order	117.6 44.4	0.27 4.2	5.0/20°C/500	200 100	[129]

Wine processing sludge	Cr(VI)	Pseudo-second order	2.42	0.070	4.2/50/240	100	[73]

Wine processing sludge	Ni(II)	Pseudo-second order	3.11	0.226	5.5/50/120	45	[76]

Desiccated coconut	Hg(II)	Pseudo-second order	447.03	—	7.4/30/60	50	[100]

Pecan nut shells (*C. illinoinensis*) biomass	Acid Blue 74 (AB74)	Pseudo-first	3.271	0.02	6.5/30/500	1000	[132]
	Reactive Blue 4 (RB4)	Pseudo-second	10.010	4.35∗10^−4^	6.5/30/1000		
	Acid Blue 25 (AB25)	Pseudo-second-order	4.892	7.15∗10^−3^	6.5/30/500		

Spent brewery grains	AG25 dye	Pseudo-second order	74.63	0.038	3.0/30/75	90	[70]

Beer brewery diatomite waste (SDE)	Methylene Blue basic dye	Pseudo-second order	4.92	1.24	7.0/25/1440	2.5	[81]

Antibiotic waste *P*. *mutilus *	Basic Blue 41	Pseudo-second order	90.91	0.0042	8.0-9.0/30/60	70	[119]

Macrofungal waste from antibiotics	Cd(II)	Pseudo-second order	82.8	0.0014	5.0/20/15	200	[120]

Fennel biomass (*Foeniculum vulgare*)	Cd(II)	Pseudo-second order	9.30	0.476	5.0/30/50	100	[123]

*Phaseolus vulgaris L. *	Reactive Red 198	Pseudo-second order	81.97	0.036	2.0/20/20	—	[86]

Nonliving biomass *Aspergillus awamori *	Cu(II)	Pseudo-first order	35.00	0.077	5.0/20/180	25	[125]

*Corynebacterium* *glutamicum *	Reactive Black 5 RB5	Pseudo-second order	370.00	9.4∗10^−5^	1.0/25/500	2000	[127]

*Phaseolus vulgaris L. *	Acid Red 57 dye	Pseudo-second order	89.49	0.21	2.0/20/20	150	[91]

Fruit waste macrofungi *Flammulina velutipes *	Cd(II)	Pseudo-first					[130]
	Pb(II)	Pseudo-second order	13.04	2.17	6.0/25/60	10	

Orange (*Citrus sinensis*)	Cr(III)	Pseudo-second order	10.97	0.002	5.0/25/4320	100	[102]

Pectin-rich fruit wastes (lemon peels)	Cd(II)	Pseudo-second order	13.92	0.021	5.0/—/50	19.2	[133]

Orange waste	Cd(II)	Elovich	333.33 (1/*α*)	0.004 (1/*β*)	6.0/25/60	100	[94]

*Mangifera indica* (mango) seed kernel particles	Methylene Blue (MB) cationic dye	Pseudo-first order	115	0.0461	8.0/30/120	175	[98]

*Rhizopus oligosporus* biomass	Cu(II)	Pseudo-second order	69.82	0.002	5.0/30/120	100	[85]

Crushed olive stone wastes	Pb(II)	Pseudo-second order	1.12	0.141	5.5/20/60	18.86	[105]
	Ni(II)		0.25	3.000		4.48	
	Cu(II)		0.26	7.497		4.35	
	Cd(II)		0.72	0.121		10.56	

Olive stones	Cd(II)	Pseudo-second order	0.903	3.196	11.0/80/20	10	[106]

Palm oil mill effluent (POME) sludge	Methylene Blue (MB) cationic dye	Pseudo-second order	5.54	0.0072	7.6/27/4320	10	[111]

Olive pomace	Methylene Blue (MB) dye	Pseudo-second order	—	0.0906	—/25/240	10	[109]

Tea industry waste	Cd(II)	Pseudo-second order	10.6	0.02	7.0/25/180	100	[72]

Pineapple peel, an agricultural effluent	Methylene Blue (MB) cationic dye	Pseudo-second order	104.17	0.22 × 10^−3^	6.0/30/400	300	[91]

Okra food waste	Cd(II) Fe(II) Zn(II)	Pseudo-second order	17.54 20.42 14.99	0.009 0.008 0.013	—/20/90	20	[88]

Activated carbon derived from exhausted olive waste cake	Lanaset Grey G	Pseudo-first order	106.4	0.0019	6.0/25/3000	150	[113]

Activated carbon from tea industry waste (TIWAC)	Cr(III)	Pseudo-second order	0.464	1.52	6.0/—/30	0.01	[115]

Sugar industry (waste bagasse)	Cd(II) Fe(II)	Pseudo-first order	—	9.3∗10^−5^ 7.2∗10^−5^	—/20/90	20	[89]

Sugarcane bagasse waste	Methylene Blue (MB)	Pseudo-second order	192.31	0.0012	8.0/25/600	200	[78]
	Gentian Violet		357.14	0.00005	8.0/25/900	300	

*Units depend on the fitting kinetic model and are indicated in Table 4.

## References

[1] Rafatullah M, Sulaiman O, Hashim R, Ahmad A (2010). Adsorption of methylene blue on low-cost adsorbents: a review. *Journal of Hazardous Materials*.

[2] Gupta VK (2009). Application of low-cost adsorbents for dye removal—a review. *Journal of Environmental Management*.

[3] Forgacs E, Cserháti T, Oros G (2004). Removal of synthetic dyes from wastewaters: a review. *Environment International*.

[4] Kyzas GZ, Fu J, Matis KA (2013). The change from past to future for adsorbent materials in treatment of dyeing wastewaters. *Materials*.

[5] Vijayaraghavan J, Basha SJS, Jegan J (2013). A review on efficacious methods to decolorize reactive azo dye. *Journal of Urban and Environmental Engineering*.

[6] Aksu Z (2005). Application of biosorption for the removal of organic pollutants: a review. *Process Biochemistry*.

[7] Wang J, Chen C (2009). Biosorbents for heavy metals removal and their future. *Biotechnology Advances*.

[8] Zhou Y-F, Haynes RJ (2010). Sorption of heavy metals by inorganic and organic components of solid wastes: significance to use of wastes as low-cost adsorbents and immobilizing agents. *Critical Reviews in Environmental Science and Technology*.

[9] Fu F, Wang Q (2011). Removal of heavy metal ions from wastewaters: a review. *Journal of Environmental Management*.

[10] Gadd GM (2009). Biosorption: critical review of scientific rationale, environmental importance and significance for pollution treatment. *Journal of Chemical Technology and Biotechnology*.

[11] Gogate PR, Pandit AB (2004). A review of imperative technologies for wastewater treatment I: oxidation technologies at ambient conditions. *Advances in Environmental Research*.

[12] Gogate PR, Pandit AB (2004). A review of imperative technologies for wastewater treatment II: hybrid methods. *Advances in Environmental Research*.

[13] Bolong N, Ismail AF, Salim MR, Matsuura T (2009). A review of the effects of emerging contaminants in wastewater and options for their removal. *Desalination*.

[14] Chojnacka K (2010). Biosorption and bioaccumulation—the prospects for practical applications. *Environment International*.

[15] Crini G (2006). Non-conventional low-cost adsorbents for dye removal: a review. *Bioresource Technology*.

[16] Ngah WSW, Teong LC, Hanafiah MAKM (2011). Adsorption of dyes and heavy metal ions by chitosan composites: a review. *Carbohydrate Polymers*.

[17] Demirbas A (2009). Agricultural based activated carbons for the removal of dyes from aqueous solutions: a review. *Journal of Hazardous Materials*.

[18] Crini G, Badot P-M (2008). Application of chitosan, a natural aminopolysaccharide, for dye removal from aqueous solutions by adsorption processes using batch studies: a review of recent literature. *Progress in Polymer Science*.

[19] Salleh MAM, Mahmoud DK, Karim WAWA, Idris A (2011). Cationic and anionic dye adsorption by agricultural solid wastes: a comprehensive review. *Desalination*.

[20] Kaushik P, Malik A (2009). Fungal dye decolourization: recent advances and future potential. *Environment International*.

[21] Pearce CI, Lloyd JR, Guthrie JT (2003). The removal of colour from textile wastewater using whole bacterial cells: a review. *Dyes and Pigments*.

[22] Sud D, Mahajan G, Kaur MP (2008). Agricultural waste material as potential adsorbent for sequestering heavy metal ions from aqueous solutions—a review. *Bioresource Technology*.

[23] Lesmana SO, Febriana N, Soetaredjo FE, Sunarso J, Ismadji S (2009). Studies on potential applications of biomass for the separation of heavy metals from water and wastewater. *Biochemical Engineering Journal*.

[24] Farooq U, Kozinski JA, Khan MA, Athar M (2010). Biosorption of heavy metal ions using wheat based biosorbents—a review of the recent literature. *Bioresource Technology*.

[25] Fosso-Kankeu E, Mulaba-Bafubiandi AF (2014). Implication of plants and microbial metalloproteins in the bioremediation of polluted waters: a review. *Physics and Chemistry of the Earth*.

[26] Ahluwalia SS, Goyal D (2007). Microbial and plant derived biomass for removal of heavy metals from wastewater. *Bioresource Technology*.

[27] Yadanaparthi SKR, Graybill D, von Wandruszka R (2009). Adsorbents for the removal of arsenic, cadmium, and lead from contaminated waters. *Journal of Hazardous Materials*.

[28] Demirbas A (2008). Heavy metal adsorption onto agro-based waste materials: a review. *Journal of Hazardous Materials*.

[29] Yan L, Yin H, Zhang S, Leng F, Nan W, Li H (2010). Biosorption of inorganic and organic arsenic from aqueous solution by *Acidithiobacillus ferrooxidans* BY-3. *Journal of Hazardous Materials*.

[30] Mathialagan T, Viraraghavan T (2005). Biosorption of pentachlorophenol by fungal biomass from aqueous solutions: a factorial design analysis. *Environmental Technology*.

[31] Maciel GM, Souza CGMD, Araújo CAVD (2013). Biosorption of herbicide picloram from aqueous solutions by live and heat-treated biomasses of *Ganoderma lucidum* (Curtis) P. Karst and *Trametes* sp.. *Chemical Engineering Journal*.

[32] Hai FI, Modin O, Yamamoto K, Fukushi K, Nakajima F, Nghiem LD (2012). Pesticide removal by a mixed culture of bacteria and white-rot fungi. *Journal of the Taiwan Institute of Chemical Engineers*.

[33] Nguyen LN, Hai FI, Yang S (2014). Removal of pharmaceuticals, steroid hormones, phytoestrogens, UV-filters, industrial chemicals and pesticides by *Trametes versicolor*: role of biosorption and biodegradation. *International Biodeterioration and Biodegradation*.

[34] Jain AK, Gupta VK, Jain S (2004). Removal of chlorophenols using industrial wastes. *Environmental Science and Technology*.

[35] Kurniawan TA, Chan GYS, Lo W-H, Babel S (2006). Comparisons of low-cost adsorbents for treating wastewaters laden with heavy metals. *Science of the Total Environment*.

[36] Bhatnagar A, Minocha AK (2006). Conventional and non-conventional adsorbents for removal of pollutants from water—a review. *Indian Journal of Chemical Technology*.

[37] Saraswat S, Rai JPN (2010). Heavy metal adsorption from aqueous solution using *Eichhornia crassipes* dead biomass. *International Journal of Mineral Processing*.

[38] Li H, Lin Y, Guan W (2010). Biosorption of Zn(II) by live and dead cells of *Streptomyces ciscaucasicus* strain CCNWHX 72-14. *Journal of Hazardous Materials*.

[39] Fu Y, Fu Y, Viraraghavan T (2000). Removal of a dye from an aqueous solution by the fungus aspergillus niger. *Water Quality Research Journal of Canada*.

[40] Fu Y, Viraraghavan T (2002). Removal of Congo Red from an aqueous solution by fungus *Aspergillus niger*. *Advances in Environmental Research*.

[41] Bishnoi NR, Pant A, Garima (2004). Biosorption of copper from aqueous solution using algal biomass. *Journal of Scientific and Industrial Research*.

[42] Navarro AE, Portales RF, Sun-Kou MR, Llanos BP (2008). Effect of pH on phenol biosorption by marine seaweeds. *Journal of Hazardous Materials*.

[43] Koçberber N, Dönmez G (2007). Chromium(VI) bioaccumulation capacities of adapted mixed cultures isolated from industrial saline wastewaters. *Bioresource Technology*.

[44] Srivastava S, Ahmad AH, Thakur IS (2007). Removal of chromium and pentachlorophenol from tannery effluents. *Bioresource Technology*.

[45] Bishnoi NR, Bajaj M, Sharma N, Gupta A (2004). Adsorption of Cr(VI) on activated rice husk carbon and activated alumina. *Bioresource Technology*.

[46] Kumar U, Bandyopadhyay M (2006). Sorption of cadmium from aqueous solution using pretreated rice husk. *Bioresource Technology*.

[47] Singh KK, Rastogi R, Hasan SH (2005). Removal of cadmium from wastewater using agricultural waste “rice polish”. *Journal of Hazardous Materials*.

[48] Tarley CRT, Arruda MAZ (2004). Biosorption of heavy metals using rice milling by-products. Characterisation and application for removal of metals from aqueous effluents. *Chemosphere*.

[49] Mohan D, Singh KP, Singh VK (2006). Chromium (III) removal from wastewater using low cost activated carbon derived from agriculture waste material and activated carbon fabric filter. *Journal of Hazardous Materials*.

[50] Mohan D, Singh KP, Singh VK (2006). Trivalent chromium removal from wastewater using low cost activated carbon derived from agricultural waste material and activated carbon fabric cloth. *Journal of Hazardous Materials*.

[51] Kumar PS, Ramalingam S, Senthamarai C, Niranjanaa M, Vijayalakshmi P, Sivanesan S (2010). Adsorption of dye from aqueous solution by cashew nut shell: studies on equilibrium isotherm, kinetics and thermodynamics of interactions. *Desalination*.

[52] Venkateswarlu P, Ratnam MV, Rao DS, Rao MV (2007). Removal of chromium from aqueous solution using *Azadirachta indica* (neem) leaf powder as an adsorbent. *International Journal of Physical Sciences*.

[53] Ahluwalia SS, Goyal D (2005). Removal of heavy metals by waste tea leaves from aqueous solution. *Engineering in Life Sciences*.

[54] Benaïssa H (2006). Screening of new sorbent materials for cadmium removal from aqueous solutions. *Journal of Hazardous Materials*.

[55] Schiewer S, Patil SB (2008). Modeling the effect of pH on biosorption of heavy metals by citrus peels. *Journal of Hazardous Materials*.

[56] Farajzadeh MA, Monji AB (2004). Adsorption characteristics of wheat bran towards heavy metal cations. *Separation and Purification Technology*.

[57] Lima IS, Ribeiro ES, Airoldi C (2006). The use of chemically modified chitosan with succinic anhydride in the methylene blue adsorption. *Quimica Nova*.

[58] Cestari AR, Vieira EFS, dos Santos AGP, Mota JA, de Almeida VP (2004). Adsorption of anionic dyes on chitosan beads. 1. The influence of the chemical structures of dyes and temperature on the adsorption kinetics. *Journal of Colloid and Interface Science*.

[59] Wang J, Chen C (2014). Chitosan-based biosorbents: modification and application for biosorption of heavy metals and radionuclides. *Bioreseource Technology*.

[60] Pinto PX, Al-Abed SR, Reisman DJ (2011). Biosorption of heavy metals from mining influenced water onto chitin products. *Chemical Engineering Journal*.

[61] Otero M, Rozada F, Calvo LF, García AI, Morán A (2003). Kinetic and equilibrium modelling of the methylene blue removal from solution by adsorbent materials produced from sewage sludges. *Biochemical Engineering Journal*.

[62] Chu HC, Chen KM (2002). Reuse of activated sludge biomass: I. Removal of basic dyes from wastewater by biomass. *Process Biochemistry*.

[63] Manu B, Chaudhari S (2002). Anaerobic decolorisation of simulated textile wastewater containing azo dyes. *Bioresource Technology*.

[64] Ramalho PA, Scholze H, Cardoso MH, Ramalho MT, Oliveira-Campos AM (2002). Improved conditions for the aerobic reductive decolourisation of azo dyes by *Candida zeylanoides*. *Enzyme and Microbial Technology*.

[65] Basibuyuk M, Forster CF (2003). An examination of the adsorption characteristics of a basic dye (Maxilon Red BL-N) on to live activated sludge system. *Process Biochemistry*.

[66] Jianlong W, Yi Q, Horan N, Stentiford E (2000). Bioadsorption of pentachlorophenol (PCP) from aqueous solution by activated sludge biomass. *Bioresource Technology*.

[67] Federici F, Fava F, Kalogerakis N, Mantzavinos D (2009). Valorisation of agro-industrial by-products, effluents and waste: concept, opportunities and the case of olive mill waste waters. *Journal of Chemical Technology and Biotechnology*.

[68] BSR Report (2013). *Analysis of U.S. Food Waste among Food Manufacturers, Retailers & Wholesalers*.

[69] Han R, Li H, Li Y, Zhang J, Xiao H, Shi J (2006). Biosorption of copper and lead ions by waste beer yeast. *Journal of Hazardous Materials*.

[70] Jaikumar V, Ramamurthi V (2009). Effect of biosorption parameters kinetics isotherm and thermodynamics for acid green dye biosorption from aqueous solution by brewery waste. *International Journal of Chemistry*.

[71] Dias MA, Rosa CA, Linardi VR, Conte RA, de Castro HF (2001). Application of factorial design to study of heavy metals biosorption by waste biomass from beverage distillery. *Applied Biochemistry and Biotechnology A: Enzyme Engineering and Biotechnology*.

[72] Lu H, Xia H Biosorption of cadmium from aqueous solution onto tea industry-waste.

[73] Liu C-C, Wang M-K, Chiou C-S, Li Y-S, Lin Y-A, Huang S-S (2006). Chromium removal and sorption mechanism from aqueous solutions by wine processing waste sludge. *Industrial and Engineering Chemistry Research*.

[74] Parvathi K, Nagendran R (2008). Functional groups on waste beer yeast involved in chromium biosorption from electroplating effluent. *World Journal of Microbiology and Biotechnology*.

[75] Dias MA, Castro HF, Pimentel PF, Gomes NCM, Rosa CA, Linardi VR (2000). Removal of heavy metals from stainless steel effluents by waste biomass from Brazilian alcoholic beverage production. *World Journal of Microbiology and Biotechnology*.

[76] Liu C-C, Kuang-Wang M, Li Y-S (2005). Removal of nickel from aqueous solution using wine processing waste sludge. *Industrial and Engineering Chemistry Research*.

[77] Pujol D, Bartrolí M, Fiol N, Torre FDL, Villaescusa I, Poch J (2013). Modelling synergistic sorption of Cr(VI), Cu(II) and Ni(II) onto exhausted coffee wastes from binary mixtures Cr(VI)-Cu(II) and Cr(VI)-Ni(II). *Chemical Engineering Journal*.

[78] Gusmão KAG, Gurgel LVA, Melo TMS, Gil LF (2013). Adsorption studies of methylene blue and gentian violet on sugarcane bagasse modified with EDTA dianhydride (EDTAD) in aqueous solutions: kinetic and equilibrium aspects. *Journal of Environmental Management*.

[79] Farinella NV, Matos GD, Lehmann EL, Arruda MAZ (2008). Grape bagasse as an alternative natural adsorbent of cadmium and lead for effluent treatment. *Journal of Hazardous Materials*.

[80] Majdik C, Burcǎ S, Mǎicǎneanu A, Stanca M, Tonk S, Mezey P (2010). Suspended and immobilized brewery waste biomass and commercial yeast as biosorbents for Cd(II) removal. A thermodynamic study. *Revue Roumaine de Chimie*.

[81] Tsai W-T, Hsu H-C, Su T-Y, Lin K-Y, Lin C-M (2008). Removal of basic dye (methylene blue) from wastewaters utilizing beer brewery waste. *Journal of Hazardous Materials*.

[82] Parvathi K, Nagendran R, Nareshkumar R (2007). Lead biosorption onto waste beer yeast by-product, a means to decontaminate effluent generated from battery manufacturing industry. *Electronic Journal of Biotechnology*.

[83] Oyelude EO, Appiah-Takyi F (2012). Removal of methylene blue from aqueous solution using alkali-modified malted sorghum mash. *Turkish Journal of Engineering and Environmental Sciences*.

[84] Özcan AS, Tunali S, Akar T, Özcan A (2009). Biosorption of lead(II) ions onto waste biomass of *Phaseolus vulgaris* L.: estimation of the equilibrium, kinetic and thermodynamic parameters. *Desalination*.

[85] Ozsoy HD, Kumbur H, Saha B, van Leeuwen JH (2008). Use of *Rhizopus oligosporus* produced from food processing wastewater as a biosorbent for Cu(II) ions removal from the aqueous solutions. *Bioresource Technology*.

[86] Akar ST, Özcan AS, Akar T, Özcan A, Kaynak Z (2009). Biosorption of a reactive textile dye from aqueous solutions utilizing an agro-waste. *Desalination*.

[87] Cardoso NF, Lima EC, Pinto IS (2011). Application of cupuassu shell as biosorbent for the removal of textile dyes from aqueous solution. *Journal of Environmental Management*.

[88] Al-Barak ABS, El-Said SM (2010). The use of some food industrial by-products for waste water purification. *Research Journal of Environmental Sciences*.

[89] Kumar A, Sahu O (2013). Sugar industry waste as removal of toxic metals from waste water. *World Journal of Chemical Education*.

[90] Tunali S, Ozcan A, Kaynak Z, Ozcan AS, Akar T (2007). Utilization of the *Phaseolus vulgaris* L. waste biomass for decolorization of the textile dye Acid Red 57: determination of equilibrium, kinetic and thermodynamic parameters. *Journal of Environmental Science and Health A: Toxic/Hazardous Substances and Environmental Engineering*.

[91] Krishni RR, Foo KY, Hameed BH (2013). Food cannery effluent, pineapple peel as an effective low-cost biosorbent for removing cationic dye from aqueous solutions. *Desalination and Water Treatment*.

[92] Lasheen MR, Ammar NS, Ibrahim HS (2012). Adsorption/desorption of Cd(II), Cu(II) and Pb(II) using chemically modified orange peel: equilibrium and kinetic studies. *Solid State Sciences*.

[93] Marín ABP, Aguilar MI, Meseguer VF, Ortuño JF, Sáez J, Lloréns M (2009). Biosorption of chromium (III) by orange (*Citrus cinensis*) waste: batch and continuous studies. *Chemical Engineering Journal*.

[94] Schiewer S, Patil SB (2008). Pectin-rich fruit wastes as biosorbents for heavy metal removal: equilibrium and kinetics. *Bioresource Technology*.

[95] Pérez-Marín AB, Zapata VM, Ortuño JF, Aguilar MI, Sáez J, Lloréns M (2007). Removal of cadmium from aqueous solutions by adsorption onto orange waste. *Journal of Hazardous Materials*.

[96] Pérez Marín AB, Ortuño JF, Aguilar MI, Meseguer VF, Sáez J, Lloréns M (2010). Use of chemical modification to determine the binding of Cd(II), Zn(II) and Cr(III) ions by orange waste. *Biochemical Engineering Journal*.

[97] Rashed MN (2006). Fruit stones from industrial waste for the removal of lead ions from polluted water. *Environmental Monitoring and Assessment*.

[98] Kumar KV, Kumaran A (2005). Removal of methylene blue by mango seed kernel powder. *Biochemical Engineering Journal*.

[99] Alencar WS, Acayanka E, Lima EC (2012). Application of *Mangifera indica* (mango) seeds as a biosorbent for removal of Victazol Orange 3R dye from aqueous solution and study of the biosorption mechanism. *Chemical Engineering Journal*.

[100] Johari K, Saman N, Song ST, Mat H, Stuckey DC (2013). Utilization of coconut milk processing waste as a low-cost mercury sorbent. *Industrial Engineering Chemistry Research*.

[101] Malkoc E, Nuhoglu Y, Dundar M (2006). Adsorption of chromium(VI) on pomace—an olive oil industry waste: batch and column studies. *Journal of Hazardous Materials*.

[102] Blázquez G, Calero M, Hernáinz F, Tenorio G, Martín-Lara MA (2010). Equilibrium biosorption of lead(II) from aqueous solutions by solid waste from olive-oil production. *Chemical Engineering Journal*.

[103] Martinez-Garcia G, Bachmann RT, Williams CJ, Burgoyne A, Edyvean RGJ (2006). Olive oil waste as a biosorbent for heavy metals. *International Biodeterioration and Biodegradation*.

[104] Vegliò F, Beolchini F, Prisciandaro M (2003). Sorption of copper by olive mill residues. *Water Research*.

[105] Fiol N, Villaescusa I, Martínez M, Miralles N, Poch J, Serarols J (2006). Sorption of Pb(II), Ni(II), Cu(II) and Cd(II) from aqueous solution by olive stone waste. *Separation and Purification Technology*.

[106] Blázquez G, Hernáinz F, Calero M, Ruiz-Núñez LF (2005). Removal of cadmium ions with olive stones: the effect of some parameters. *Process Biochemistry*.

[107] Pagnanelli F, Mainelli S, Vegliò F, Toro L (2003). Heavy metal removal by olive pomace: biosorbent characterisation and equilibrium modelling. *Chemical Engineering Science*.

[108] Fernando A, Monteiro S, Pinto F, Mendes B (2009). Production of biosorbents from waste olive cake and its adsorption characteristics for Zn^2+^ ion. *Sustainability*.

[109] Banat F, Al-Asheh S, Al-Ahmad R, Bni-Khalid F (2007). Bench-scale and packed bed sorption of methylene blue using treated olive pomace and charcoal. *Bioresource Technology*.

[110] Stasinakis AS, Elia I, Petalas AV, Halvadakis CP (2008). Removal of total phenols from olive-mill wastewater using an agricultural by-product, olive pomace. *Journal of Hazardous Materials*.

[111] Abbas M, Zaini A, Zakaria M, Setapar SHM, Yunus MAC (2013). Sludge-adsorbents from palm oil mill effluent for methylene blue removal. *Journal of Environmental Chemical Engineering*.

[112] Zhang Y, Banks C (2006). A comparison of the properties of polyurethane immobilised Sphagnum moss, seaweed, sunflower waste and maize for the biosorption of Cu, Pb, Zn and Ni in continuous flow packed columns. *Water Research*.

[113] Baccar R, Blánquez P, Bouzid J, Feki M, Sarrà M (2010). Equilibrium, thermodynamic and kinetic studies on adsorption of commercial dye by activated carbon derived from olive-waste cakes. *Chemical Engineering Journal*.

[114] Foo KY, Hameed BH (2011). Preparation of oil palm (Elaeis) empty fruit bunch activated carbon by microwave-assisted KOH activation for the adsorption of methylene blue. *Desalination*.

[115] Duran C, Ozdes D, Gundogdu A, Imamoglu M, Senturk HB (2011). Tea-industry waste activated carbon, as a novel adsorbent, for separation, preconcentration and speciation of chromium. *Analytica Chimica Acta*.

[116] Karthika C, Vennilamani N, Pattabhi S, Sekar M (2010). Utilization of sago waste as an adsorbent for the removal of Pb(II) from aqueous solution: kinetic and isotherm studies. *International Journal of Engineering Science and Technology*.

[117] Guo C, Yue T, Yuan Y (2013). Biosorption of patulin from apple juice by caustic treated waste cider yeast biomass. *Food Control*.

[118] Svecova L, Spanelova M, Kubal M, Guibal E (2006). Cadmium, lead and mercury biosorption on waste fungal biomass issued from fermentation industry. I. Equilibrium studies. *Separation and Purification Technology*.

[119] Yeddou-Mezenner N (2010). Kinetics and mechanism of dye biosorption onto an untreated antibiotic waste. *Desalination*.

[120] Moussous S, Selatnia A, Merati A, Junter GA (2012). Batch cadmium(II) biosorption by an industrial residue of macrofungal biomass (*Clitopilus scyphoides*). *Chemical Engineering Journal*.

[121] Prigione V, Grosso I, Tigini V, Anastasi A, Varese GC (2012). Fungal waste-biomasses as potential low-cost biosorbents for decolorization of textile wastewaters. *Water*.

[122] Rao KR, Rashmi K, Latha JNL, Mohan PM (2005). Bioremediation of toxic metal ions using biomass of *Aspergillus fumigatus* from fermentative waste. *Indian Journal of Biotechnology*.

[123] Rao RAK, Khan MA, Rehman F (2010). Utilization of Fennel biomass (*Foeniculum vulgari*) a medicinal herb for the biosorption of Cd(II) from aqueous phase. *Chemical Engineering Journal*.

[124] Gochev VK, Velkova ZI, Stoytcheva MS (2010). Hexavalent chromium removal by waste mycelium of *Aspergillus awamori*. *Journal of the Serbian Chemical Society*.

[125] Velkova Z, Stoytcheva M, Gochev V (2012). Biosorption of Cu (II) onto chemically modified waste mycelium of *Aspergillus awamori*: equilibrium, kinetics and modeling studies. *Journal of Bioscience & Biotechnology*.

[126] Budinova T, Petrov N, Parra J, Baloutzov V (2008). Use of an activated carbon from antibiotic waste for the removal of Hg(II) from aqueous solution. *Journal of Environmental Management*.

[127] Vijayaraghavan K, Yun Y-S (2007). Utilization of fermentation waste (*Corynebacterium glutamicum*) for biosorption of Reactive Black 5 from aqueous solution. *Journal of Hazardous Materials*.

[128] Fomina M, Gadd GM (2014). Biosorption: current perspectives on concept, definition and application. *Bioresource Technology*.

[129] Sassi M, Bestani B, Said AH, Benderdouche N, Guibal E (2010). Removal of heavy metal ions from aqueous solutions by a local dairy sludge as a biosorbant. *Desalination*.

[130] Zhang D, He H, Li W, Gao T, Ma P (2010). Biosorption of cadmium(II) and lead(II) from aqueous solutions by fruiting body waste of fungus *Flammulina velutipes*. *Desalination and Water Treatment*.

[131] Göksungur Y, Üren S, Güvenç U (2005). Biosorption of cadmium and lead ions by ethanol treated waste baker’s yeast biomass. *Bioresource Technology*.

[132] Aguayo-Villarreal IA, Ramírez-Montoya LA, Hernández-Montoya V, Bonilla-Petriciolet A, Montes-Morán MA, Ramírez-López EM (2013). Sorption mechanism of anionic dyes on pecan nut shells (*Carya illinoinensis*) using batch and continuous systems. *Industrial Crops and Products*.

[133] Schiewer S, Balaria A (2009). Biosorption of Pb^2+^ by original and protonated citrus peels: equilibrium, kinetics, and mechanism. *Chemical Engineering Journal*.

[134] Andreea S (2013). Brewer’s yeast: an alternative for heavy metal biosorption from waste waters. *ProEnvironment*.

[135] Keskinkan O, Goksu MZL, Yuceer A, Basibuyuk M, Forster CF (2003). Heavy metal adsorption characteristics of a submerged aquatic plant (*Myriophyllum spicatum*). *Process Biochemistry*.

[136] Xie DD, Liu YY, Wu CL, Fu JK, Xue R (2003). Studies on biosorption of Pd^2+^ by the immobilized *Saccharomyces cerevisiae* waste biomass. *Microbiology*.

[137] Dhankhar R, Hooda A (2011). Fungal biosorption—an alternative to meet the challenges of heavy metal pollution in aqueous solutions. *Environmental Technology*.

[138] Ahalya N, Ramachandra TV, Kanamadi RD (2003). Biosorption of heavy metals. *Research Journal of Chemistry and Environment*.

[139] Foo KY, Hameed BH (2010). Insights into the modeling of adsorption isotherm systems. *Chemical Engineering Journal*.

[140] Njikam E, Schiewer S (2012). Optimization and kinetic modeling of cadmium desorption from citrus peels: a process for biosorbent regeneration. *Journal of Hazardous Materials*.

